# Determination of absolute expression profiles using multiplexed miRNA analysis

**DOI:** 10.1371/journal.pone.0180988

**Published:** 2017-07-13

**Authors:** Yunke Song, Duncan Kilburn, Jee Hoon Song, Yulan Cheng, Christopher T. Saeui, Douglas G. Cheung, Carlo M. Croce, Kevin J. Yarema, Stephen J. Meltzer, Kelvin J. Liu, Tza-Huei Wang

**Affiliations:** 1 Biomedical Engineering Department, Johns Hopkins University, Baltimore, Maryland, United States of America; 2 Circulomics Inc, Baltimore, Maryland, United States of America; 3 Department of Medicine (GI Division) and Sidney Kimmel Comprehensive Cancer Center, The Johns Hopkins University School of Medicine, Baltimore, Maryland, United States of America; 4 Department of Cancer Biology and Genetics, The Ohio State University, Columbus, Ohio, United States of America; 5 Mechanical Engineering Department, Johns Hopkins University, Baltimore, Maryland, United States of America; 6 Sidney Kimmel Comprehensive Cancer Center, Johns Hopkins University, Baltimore, Maryland, United States of America; 7 Center of Cancer Nanotechnology Excellence, Johns Hopkins University, Baltimore, Maryland, United States of America; Institut de Biologie Moleculaire et Cellulaire, FRANCE

## Abstract

Accurate measurement of miRNA expression is critical to understanding their role in gene expression as well as their application as disease biomarkers. Correct identification of changes in miRNA expression rests on reliable normalization to account for biological and technological variance between samples. Ligo-miR is a multiplex assay designed to rapidly measure absolute miRNA copy numbers, thus reducing dependence on biological controls. It uses a simple 2-step ligation process to generate length coded products that can be quantified using a variety of DNA sizing methods. We demonstrate Ligo-miR’s ability to quantify miRNA expression down to 20 copies per cell sensitivity, accurately discriminate between closely related miRNA, and reliably measure differential changes as small as 1.2-fold. Then, benchmarking studies were performed to show the high correlation between Ligo-miR, microarray, and TaqMan qRT-PCR. Finally, Ligo-miR was used to determine copy number profiles in a number of breast, esophageal, and pancreatic cell lines and to demonstrate the utility of copy number analysis for providing layered insight into expression profile changes.

## Introduction

MicroRNA (miRNA) are short (18–24 nt) non-coding RNA molecules that regulate gene expression. They exert control over a wide variety of cellular processes, ranging from differentiation to growth and senescence [1–5]. Although over 2000 human miRNA are predicted to exist, individual studies often focus on smaller subsets of 5–50 miRNA that are the presumed critical players in a specific disease or cellular process [3,6–8]. These miRNA are typically identified by differential expression analysis using highly multiplexed array methods [9–12] or RNA sequencing [13–15]. Validation of identified miRNA is then performed using a higher sensitivity detection platform such as RT-qPCR [16–18]. However, screening large numbers of samples across even moderate numbers of miRNA using qRT-PCR quickly becomes cost- and time-prohibitive due to the large numbers of individual reactions that must be performed. Microarray and sequencing can provide large amounts of data but are slow and expensive when only targeted panels are needed.

One additional challenge in miRNA profiling is that quantification bias can lead to both systematic and random variability in expression data. Such variability can manifest across data from different analytical methods, research groups, users, experimental designs, and sample preparation methods. The most common approach to reduce this variability is through normalization. miRNA assays are typically performed using a constant amount of total RNA as input. The resulting expression is then normalized to biological controls [19–23], such as small nucleolar RNA (e.g., RNU44, RNU6B) or stably expressed miRNA (e.g., miR-16, let-7a), or to spiking controls (e.g., cel-miR-39) to account for technical variability and variations in baseline transcriptional state. While sources of technical variability are more easily predicted and accounted for using spike-in controls, sources of biological variability are difficult to pinpoint and choosing such internal controls can be quite challenging. Normalization controls can dramatically affect expression profiles and potentially account for much of the reported variability in differential expression profiles, especially for miRNA expressed at low levels with subtle differential changes (<2-fold).

Controls are often chosen empirically by identifying miRNAs that exhibit the lowest variability across a given sample set [22] or those which most closely track the global mean of miRNA expression [23]. Both of these approaches have a central problem: empirically measured parameters—variability, global mean—cannot be measured without proper normalization. Choosing to normalize to a miRNA that exhibits lowest variability is similar to leaving the data un-normalized. Normalization that does not accurately track sources of biological and experimental variability, as may occur based on empirical selection without an understanding of underlying function, can amplify variability and generate artificial trends. RNU44 is commonly, if controversially [20], used as a biological control. Additionally, the global mean expression value has been reported to be a good normalization control [19,21], but this parameter relies on sufficient miRNAs being measured to obtain a reasonable global mean. Exogenous spike in controls have recently been shown to be a reliable normalizer for circulating miRNAs [24,25], particularly when identifying miRNAs with small expression changes [24].

Normalization to reduce methodological errors may also be combined with other approaches, such as adopting the standard practices of the miRQC guidelines [26]. Yet, the most robust approach may be the direct determination of absolute copy number. Unfortunately with existing RT-qPCR, microarray, and sequencing methods, accurate determination of absolute expression (as opposed to relative expression) is tedious and challenging. Digital PCR has been used to determine absolute miRNA expression in human serum [27–29]. While digital PCR is sensitive enough to detect even single copies with high repeatability [27], the high cost and complexity make it difficult to screen large numbers of samples across large panels of miRNA.

We have developed a flexible, rapid, and easy-to-use assay called Ligo-miR that can accurately determine miRNA copy number [30]. Ligo-miR uses a multiplex ligation process to generate length-coded, fluorescently-labeled DNA products. The simple length coding enables quantification using a wide variety of DNA sizing methods. Herein, we combine Ligo-miR with polyacrylamide gel electrophoresis (PAGE) detection in a variant called Ligo-miR EZ to perform 26-plex miRNA profiling using only a thermal cycler and PAGE apparatus. This equipment is readily available in nearly all molecular biology labs and makes Ligo-miR EZ ideally suited to repeated profiling of large numbers of samples once a targeted panel has been identified. Minor modifications to the core workflow can be made to custom tailor sensitivity, multiplex capability, and sample throughput based on application and detection methodology. For example, capillary electrophoresis (CE) and single molecule separation [31] may be used instead of PAGE to further enhance multiplex capability and sensitivity.

Ligo-miR EZ's optimized 2-step ligation mechanism enables high multiplex capability and high specificity discrimination of closely-related miRNA species. Specificity studies demonstrate high specificity discrimination of miRNA family members with single nucleotide differences and absolute discrimination of precursor and mature miRNA. A linear amplification, as opposed to exponential amplification, is performed during the 2nd ligation step to enhance sensitivity while minimizing amplification variability and bias for high reproducibility and high differential sensitivity. Analysis of synthetic miRNA, cell lines, and tissue samples show that Ligo-miR EZ is capable of 20 copies per cell sensitivity with linearity across 4.5 orders of magnitude. Analysis of mock expression panels demonstrates that differential sensitivity as low as <1.2 fold is reliably achieved. In direct comparisons to microarray and RT-qPCR, Ligo-miR EZ shows high correlations with *r*^2^>0.9. Finally, we use Ligo-miR EZ to determine miRNA copy number in 3 breast cell lines and 5 esophageal cell lines and to quantify the effects of gemcitabine on metastatic pancreatic cancer cells. This system enables direct comparison of copy number expression profiles against relative expression profiles, through which we see that factors such as cell size and baseline transcription can contribute significant variability to expression data.

In addition to reducing bias and variability in miRNA expression profiles, measurement of absolute expression enables the determination of metrics such as accurate rank ordering of expression and comparison of absolute changes in expression. These metrics, alongside the more commonly measured differential changes or proportional quantification, open up an array of new tools for comparing expression profiles across patients, samples, and disease model systems.

## Materials and methods

### Cell line samples

Primary, normal, non-immortalized esophageal epithelial cells (HEEPIC), along with esophageal cancer cell lines (SKGT4 and OE33), were purchased from ScienCell Research Laboratories (Carlsbad, California, USA) and Sigma Chemical (St Louis, Missouri, USA), respectively. The Barrett’s esophageal cell lines (CHTRT and QHTRT) were generous gifts of Dr. Peter Rabinovitch, Fred Hutchinson Cancer Center.

Breast cell lines (MCF-7, MCF-10A, and MDA-MB-231) and the metastatic pancreatic cancer cell line were purchased from ATCC (Manassas, VA).

Details of cell culturing protocols can be found in S1 File.

### Total RNA/Small RNA fraction preparation

Total RNA was isolated from the esophageal cell lines using RNeasy kits (Qiagen, Valencia, CA), combined with RNase-free DNase (Qiagen, #79254), with TRIzol reagent (Life Technologies, Carlsbad, CA) used instead of the QIAzol. For the RT-qPCR and microarray experiments, this total RNA was used directly. For Ligo-miR EZ analysis, the small RNA fraction was isolated from this total RNA using both miRNeasy (Qiagen, Valencia, CA) and mirPremier (Sigma-Aldrich, St. Louis, MO) kits. The small RNA fraction was isolated directly from the breast cell lines using miRNeasy and mirPremier kits. Total RNA was isolated from the metastatic pancreatic cancer cells using miRNeasy kits. Pancreatic total RNA was purchased from Ambion (FirstChoice Human Pancreas RNA, catalog # AM7954).

### miRNA and probe synthesis

All RNA and DNA oligonucleotides (S1 File, Tables S2-S4) were synthesized by Integrated DNA Technologies (Coralville, IA) and re-suspended to give 100 μM stocks. All synthetic miRNA were further diluted to 1 μM concentration in TE buffer (10 mM Tris-HCl, pH = 8.0, 0.1 mM EDTA) and stored at -80°C. These aliquots were used to prepare lower concentration stocks and discarded after single use to guard against degradation. Further details on miRNA and probe handling protocols can be found in S1 File.

### Ligo-miR EZ assay

In the capture ligation step, the RNA sample was added to a mastermix containing the adenylated adapter, T4 RNA ligase, and buffer and incubated at 25°C for 1 hour. Then in the coding ligation, a 2nd mastermix containing the common probe, 26-plex discrimination probes, 9°N DNA ligase, and buffer was added to the 1st step products and thermal cycled for 50 cycles. Finally, the 2nd step reaction mixture was analyzed using 15% denaturing urea PAGE gel and scanned using a GE Typhoon 9410 multi-mode imager. The fluorescent gel images were analyzed using either HandyBand software (Circulomics Inc) or using a combination [32] of ImageQuant (GE Healthcare) and OriginPro (OriginLab). Specific details can be found in S1 File.

### RT-qPCR and microarray assays

Single tube Applied Biosystems TaqMan microRNA Assays, Applied Biosystems TaqMan MicroRNA Reverse Transcription Kit, and Bio-Rad iQ Supermix were used for the RT-qPCR assays. Assays were run on an Applied Biosystems 7900HT Real-Time PCR System. 5 ng of total RNA was used as input for each RT reaction and performed according to the manufacturer’s protocol.

Microarray analysis was performed by the JHMI Deep Sequencing and Microarray Core using Agilent Human miRNA Microarray Kit Release 19.0, 8x60K (G4872A, Agilent Technologies, Santa Clara, CA) following manufacturer’s protocols.

Full details of RT-qPCR and Microarray protocols can be found in S1 File.

## Results and discussion

### Assay principle

**Fig 1:**
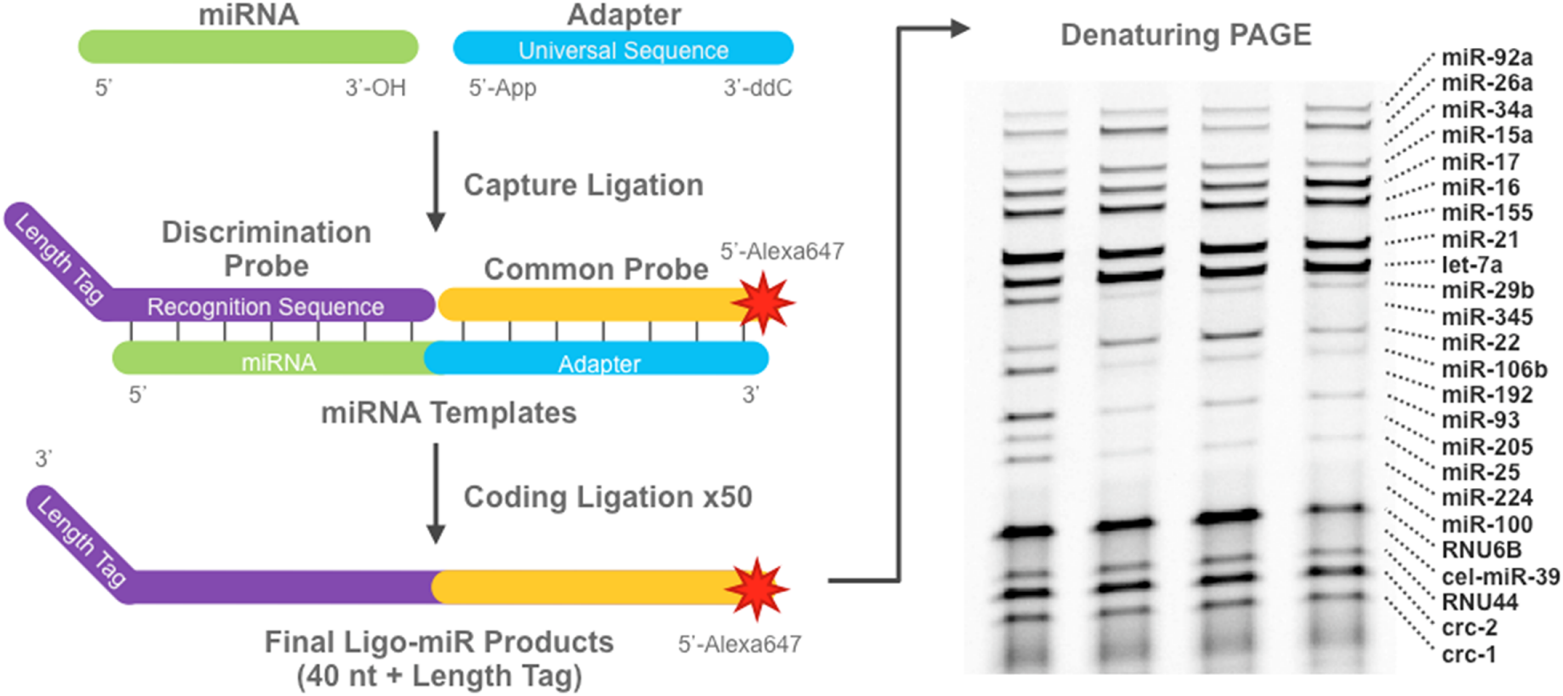
Schematic illustration of the Ligo-miR EZ multiplex miRNA assay. In the capture ligation, a universal adapter is ligated to each miRNA to form a template molecule. In the coding ligation, a miRNA specific discrimination probe and common probe are hybridized to each template and ligated together to form a single stranded Ligo-miR product. Thermal cycling is used to generate up to 50 products from a single template. Finally, the Ligo-miR products are analyzed by denaturing PAGE. Shown are 4 samples analyzed using a 24-plex Ligo-miR EZ probe set. Each band is a specific miRNA product where band intensity is proportional to quantity.

Ligo-miR EZ uses a 2-step ligation mechanism schematically illustrated in Fig 1. In the capture ligation step, a universal adapter is ligated to the 3' end of all sample miRNA to form templates. Reaction conditions and adapter design have been optimized previously [32] to ensure high efficiency (86%) and low capture bias (10% CV). Then in the coding ligation step, up to 26 miRNA specific discrimination probes (DPs) and an Alexa647-labeled common probe (CP) are hybridized to the miRNA templates from the first step and ligated together to form a single stranded DNA product. Each DP contains a recognition sequence at the 5' end that is complementary to the miRNA being detected and a length tag at the 3' end to yield a specific length product for each miRNA species. The CP sequence is complementary to the adapter probe and not miRNA specific. Thermal cycling is used to perform a 50X linear amplification by repeatedly generating Ligo-miR EZ products from each miRNA template. The Ligo-miR EZ products are then analyzed by polyacrylamide gel electrophoresis (PAGE). Circulomics HandyBand software is used to analyze the resultant gel image and determine miRNA expression based on band position and intensity.

### Sensitivity and bias

**Fig 2:**
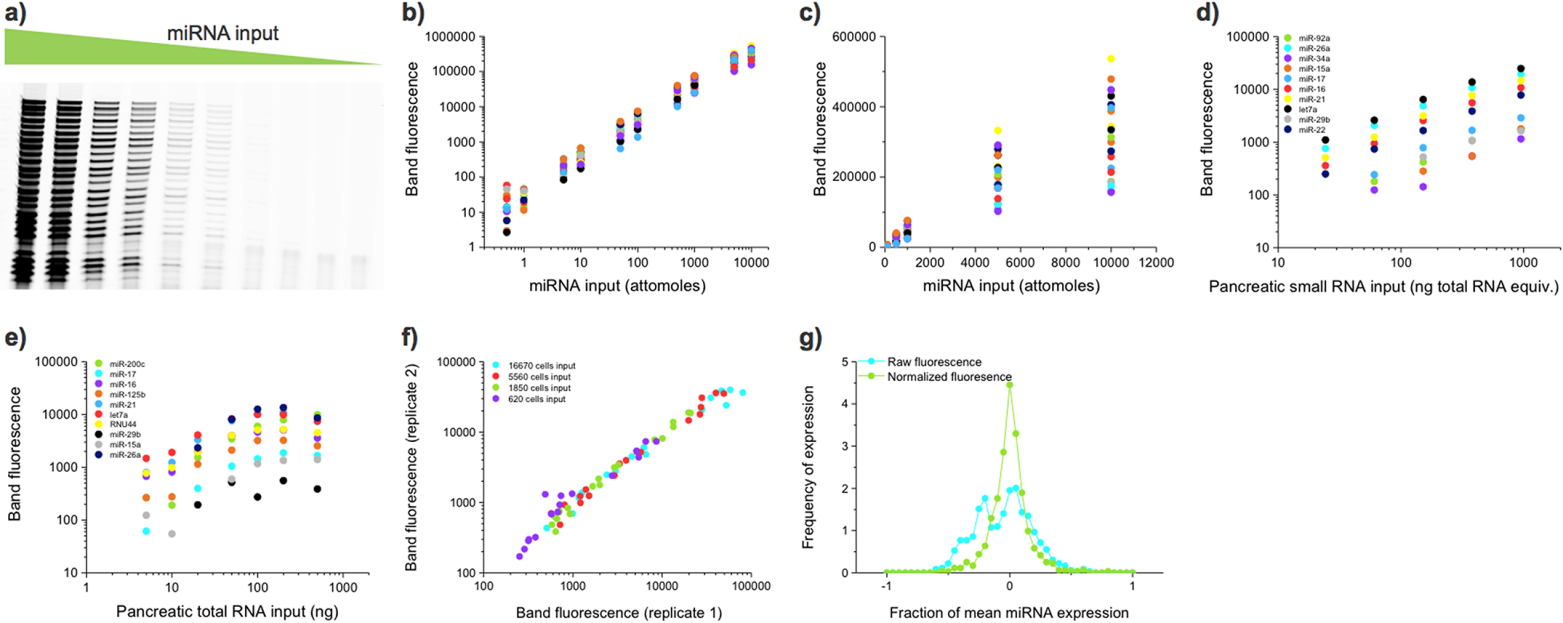
Ligo-miR EZ sensitivity and reproducibility data. **a)** A PAGE image of Ligo-miR EZ products from a 26-plex serial dilution of synthetic miRNAs from 10,000 attomoles to 0.5 attomoles. Data from 3 miRs with higher sensitivity cutoffs are omitted. Raw band fluorescent intensities are tabulated in S1 File, Table S1. **b)** Image analysis of the fluorescent band intensities. Some plateauing of the assay can be seen at the highest input level. Below this, the assay shows a linear response over 4 orders of magnitude for 12 of the 22 miRNAs (not counting Crc-1,2,3 or 4) the lowest point is missing or an outlier for 3 miRs, giving a measured dynamic range of 3.5 orders of magnitude. The lowest dynamic range is for RNU6B, we suspect because of an outlier measurement, and is only 1.5 orders of magnitude. The mean dynamic range is 3.5, the median dynamic range is 4 and the mode dynamic range is 4 orders of magnitude. **c)** Same data as shown in b) but plotted on linear-linear axes. **d**) Ligo-miR EZ was performed on a dilution series of small RNA fractions isolated from pancreatic total RNA. A linear response was seen up 1000 ng of total RNA equivalent input. **e)** When total RNA is input directly, a linear response is seen up until 100 ng at which point background RNA begins inhibiting ligation efficiency. **f)** Replicate experiments were performed on 4 samples of small RNA isolated from 600–17000 cultured cells and plotted against one another. High correlation was seen between replicate experiments across all input levels. **g)** Ligo-miR EZ was repeated 28 times on a 26-plex synthetic miRNA panel over a period of 3 months. The distribution of raw fluorescence values as a fraction of the mean is centered around zero with a side peak at -0.2. When the raw fluorescence values are corrected for scanner variability, the data have a very sharp distribution centered around the mean.

Serial dilutions of synthetic miRNA, pancreatic small RNA, and pancreatic total RNA were used to determine the assay sensitivity, dynamic range, linearity, and bias. Fig 2a shows an image of the gel resulting from a 26-plex Ligo-miR EZ analysis performed on a serial dilution of synthetic miRNA. The assay shows a linear response between miRNA input and band fluorescence over several orders of magnitude and a sensitivity as low as 0.5 attomoles when scanned using a high sensitivity imager such as GE's Typhoon 9410 (Fig 2b and S1 File, Table S1). At standard input levels, this translates to approximately 20–500,000 copies per cell, overlapping expected cellular expression levels. When rigorously performed, gel quantification and image analysis can be highly sensitive and repeatable, capable of measurement CVs <10% and sensitivity <5 attomoles of fluorophore [32].

The response from a serial dilution of small RNA isolated from pancreatic total RNA is shown in Fig 2d. However, below 75 ng of total RNA equivalent input, lowly expressed miRNA begin dropping below the sensitivity threshold, reducing the number of detectable bands. When total RNA is used as sample input as opposed to small RNA, inhibition can be seen at inputs >100 ng (Fig 2e). Rising levels of background RNA inhibit the coding ligation efficiency, offsetting signal increase and leading to a plateau in response. At each given input level, the degree of inhibition varies with miRNA species, but is repeatable and can be effectively normalized using reference curves obtained with MS2 phage RNA (S1 File, Fig S1). It is important to note that this inhibition does not reduce individual miRNA response curve linearity or dynamic range but can reduce overall signal intensity impacting sensitivity.

Amplification bias has been minimized by optimizing reaction conditions to suppress ligation bias [32,33] and by optimizing probe design via thermodynamic analysis. Amplification bias can be determined by analyzing the spread in the fluorescent intensity across miRNA. Across the 56 miRNA tested in total, no systematic trends were observed with factors such as probe T_m_, miRNA GC content, or miRNA 3’ base (S1 File, Fig S2). However, predictable differences were seen due to tag length (S1 File, Fig S3). After optimization, the raw band fluorescence for given miRNA input, as seen in Fig 2b and S1 File, Fig S9, varies less than 10X max-min across a typical Ligo-miR EZ panel.

### Reproducibility and robustness

Assay reproducibility was first examined by comparing the raw band intensities for replicate experiments at varying cell inputs. Ligo-miR EZ was highly reproducible at both high (*r*^2^ = 0.98) and low (*r*^2^ = 0.93) input levels (Fig 2f). For a long—term test of repeatability, we created a synthetic panel containing 1500 attomoles of each of 26 miRNAs. 28 repeats of this experiment spanning a period of 3 months generated an inter-day mean CV of 22% for the raw band fluorescence (S1 File, Fig S4) and 13% when normalized to miR-16-5p. Fig 2g shows the distribution of all measurements when normalized against the mean for each miRNA. For a perfect assay this distribution would be a delta function at 0 –i.e. no deviation from the mean. The distribution of the raw deviations is centered around 0, but there is a prominent side peak centered at -0.2 due to drift in scanner performance. When the band intensities were normalized, the distribution became tight and symmetric. A separate analysis determined that the intraday mean CVs of raw and normalized band intensities from 1500 attomoles were 7.9% and 5.8%, respectively (S1 File, Fig S5).

When Ligo-miR EZ was used to compare MCF-7 total RNA and small RNA inputs (S1 File, Fig S6), high correlation (*r*^2^ = 0.96) was seen. However, higher signal intensities were seen with small RNA than total RNA. High correlation was also seen when comparing small RNA isolated with either Qiagen miRNeasy or Sigma mirPremier kits (S1 File, Fig S7). Interestingly, two of the outlier data points were due to RNU44, which at 61 nts is considerably longer than miRNA. We hypothesize that this outlier may arise due to length dependent biases from different small RNA isolation chemistries employed by the two kits. This discrepancy underscores the inherent risk in using normalization controls that differ in length or structure from the miRNA being measured [22,23]. In aggregate, these data demonstrate that Ligo-miR EZ is very reproducible and robust across common sample types and sample preparation methods.

### Specificity

**Fig 3:**
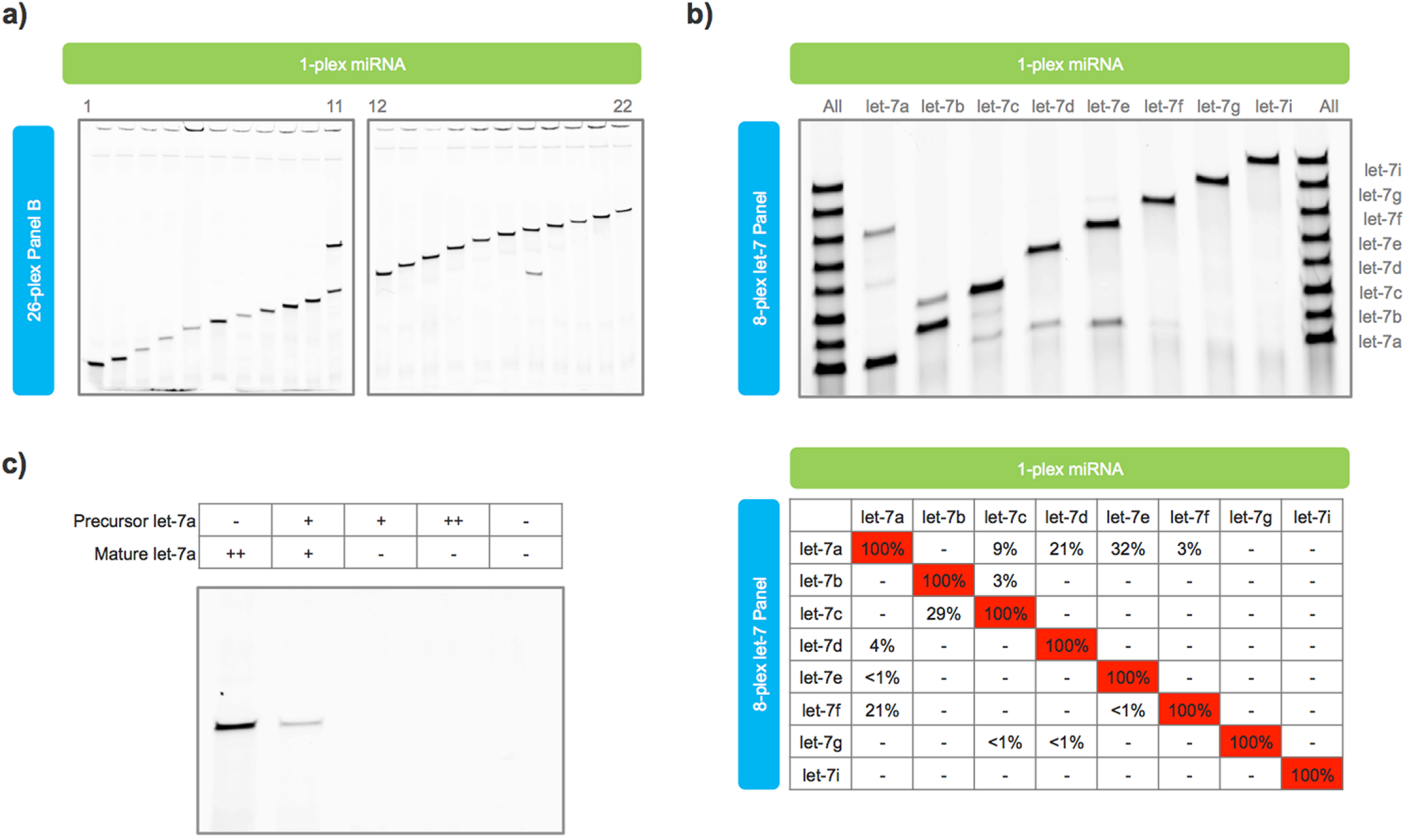
Ligo-miR EZ specificity data. **a)** Single-plex synthetic miRNAs were analyzed using Ligo-miR EZ Panel B. No cross-talk is seen across unrelated miRNA. Cross-talk is only seen in Lanes 11 and 18 which represent miR-17-5p and miR-106a-5p that differ by only a single nucleotide at the 5’ end. **b)** An 8-plex let-7 family probe set was used to test assay specificity. Lanes 1 (left) and 10 (right) contain all 8-plex miRNA while Lanes 2–9 (l—r) have 1-plex miRNAs. Limited cross-talk is seen across probe-miRNA pairs with sequence mismatches distant to the ligation site. **c)** Let-7a precursor and mature miRNA were input into a single-plex let-7a assay. No response was seen even with a high background of precursor molecules. ++ = 1500 attomoles, + = 150 attomoles,— = no miRNA.

High assay specificity is particularly important in miRNA profiling as hundreds of different miRNA can be present in any single sample. This diversity often includes families of miRNA with closely-related sequences, isomiRs with minor 5' and 3' modifications, and transcripts with mature, precursor, and primary forms. Synthetic miRNA and probe sets were designed to test assay specificity. Across unrelated miRNA, Ligo-miR EZ exhibited perfect discrimination with no detectable cross-talk (Fig 3a). Cross-talk was only observed between the miRNAs in lanes 11 and 18, which correspond to miR-106a-5p and miR-17-5p. These miRNA both come from the miR-17 precursor family and differ by only a single nucleotide at the 5’ end (S1 File, Table S2).

To test closely-related miRNA, we designed a probe set to profile 8 members of the let-7 family, 7 of which differ by only 1 or 2 nts (S1 File, Table S3). Limited cross-talk was seen in 12 of 56 off miRNA combinations (Fig 3b). Cross-talk greater than 5% was only seen in 5 probe:miRNA combinations: let-7f:let-7a, let-7c:let-7b, let-7a:let-7c, let-7a:let-7d, and let-7a:let-7e. Ligo-miR EZ is expected to be more sensitive to probe:miRNA mismatches near the ligation site due to the combination of thermodynamic mismatch discrimination and ligase mismatch discrimination. Such behavior is common to ligation assays [34–36] as mismatches far from the ligation site no longer sit within the ligase's active site [35], leaving thermodynamic discrimination as the remaining force. Thus, it was not unexpected that the greatest degree of cross-talk was seen between probe:miRNA pairs where the mismatch occurred far from the ligation site such as let-7a:let-7e (14 bases), let-7a:let-7d (7 bases), let-7c:let-7b (6 bases), and let-7f:let-7a (11 bases). Similar overall levels of cross-talk are seen in other hybridization assays [26], though some of these studies relied on earlier miRBase annotations that contained let-7 miRNA of differing lengths [16].

Across precursor and mature miRNA, Ligo-miR EZ exhibited absolute discrimination. Precursor let-7a (S1 File, Table S4) and mature let-7a miRNA were mixed in different ratios and analyzed using a single-plex let-7a discrimination probe. Even in a high background of precursor molecules, no cross-talk was seen (Fig 3c). As seen above, the 2-step ligation mechanism renders the assay extremely sensitive to variations at the miRNA 3' end. Modifications close to this end will be discriminated with high specificity while insertions or deletions will be discriminated with absolute specificity.

### Differential sensitivity

Many applications of miRNA profiling involve measuring differential expression to identify up- or down-regulated miRNAs. To quantify differential expression sensitivity, we made 4 samples (S1, S2, S3 and S4) containing 26 synthetic miRNA (4 internal controls, 20 miRNAs, 2 snRNAs) in a manner analogous to the miRQC study [26]. Base samples S1 and S2 were titrated to generate samples S3 = 0.25*S1 + 0.75*S2 and S4 = 0.75*S1 + 0.25*S2. This enabled differential sensitivity testing at low (10 attomoles), medium (100 attomoles), and high (1000 attomoles) expression levels with maximum fold-changes of 3, 3, and 2, respectively (S1 File, Fig S8). The smallest ratio between two bands is the 1.14-fold difference between S3 and S2 at the 1000 attomoles level.

**Fig 4:**
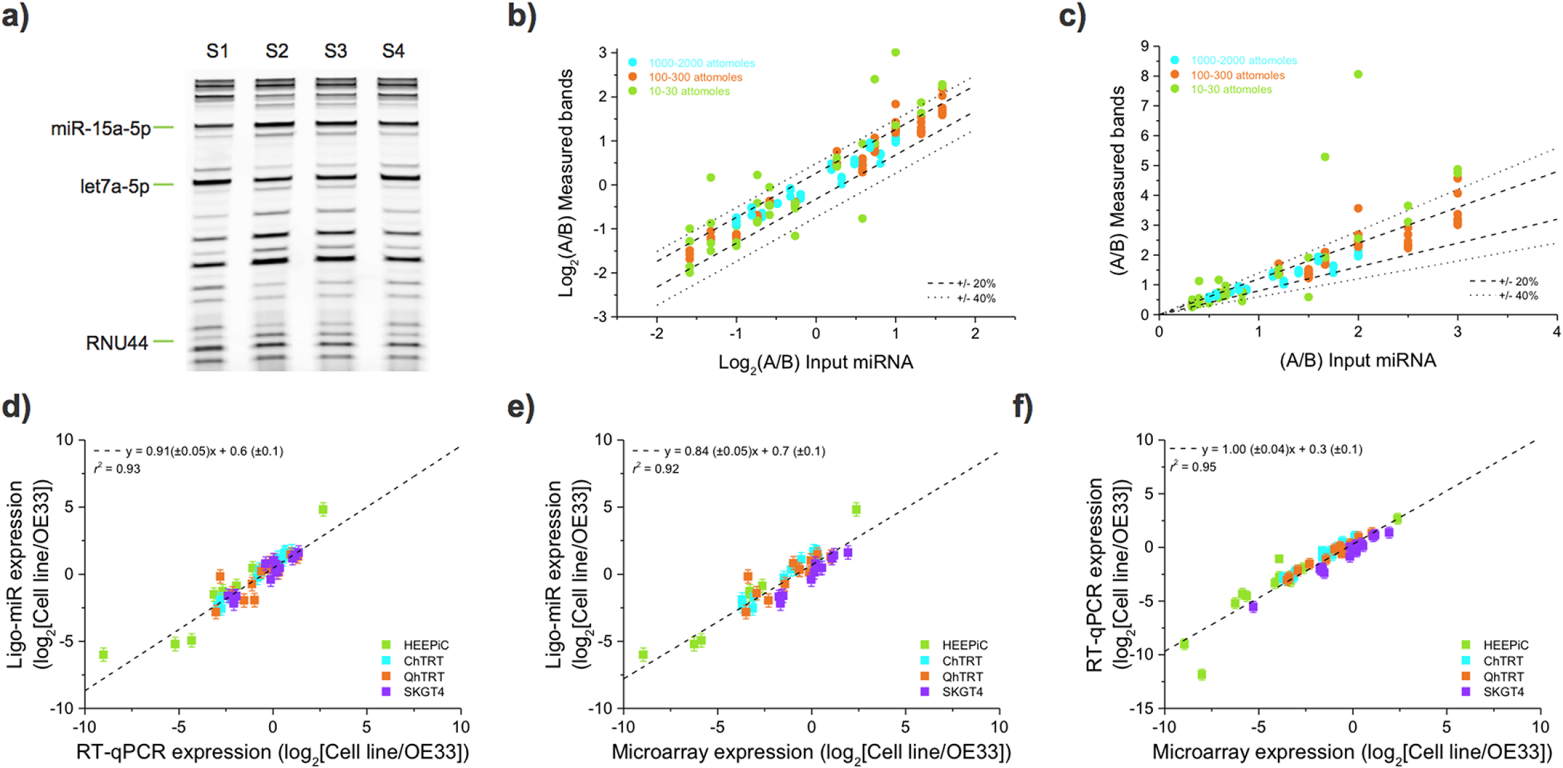
Differential sensitivity and benchmarking of Ligo-miR EZ. **a)** PAGE image of Ligo-miR EZ assay performed on S1-S4 synthetic miRNA panels. Three bands are highlighted to show how band intensities change with input level. All bands are detected but low intensity bands are difficult to visualize due to the limited contrast of the print image. **b)** The ratio of measured band intensities is plotted as a function of expected input ratios. The ratios were determined from the mean of 2 measurements. The three input levels are plotted: high (blue), medium (orange) and low (green). If the measured ratio is exactly equal to the input ratio then the resultant data will fall along the line y = x. For reference, the dotted lines illustrate where the measured ratio varies by ± 20% and ± 40% from the expected ratio. **c)** Same data as shown in b) but plotted on linear-linear axes. **d), e)** and **f)** Benchmarking was performed by using Ligo-miR EZ, TaqMan qRT-PCR, and microarray to perform differential expression analysis of 5 esophageal cell lines, with expression was compared to OE33 in all cases. All platforms showed good correlation to one another.

Ligo-miR EZ was then performed on each of the 4 samples in duplicate. A gel image from one replicate is shown in Fig 4a. We found that 19 of the 22 miRNA, or 90.5%, displayed the correct titration response (S1 < S4 < S3 < S2). For example, the band intensities for miR-15a-5p increase as expected. This measure of differential sensitivity is particularly stringent because it requires correctly resolving differences as small as 1.14-fold and no larger than 3-fold for every miRNA tested. Fig 4b and 4c plots the measured differential ratio against the expected ratio. The data are tightly packed around the correct differential response, *y* = *x*. The measured differential ratio differs from the expected ratio by an average of 9.7%, 13.4%, and 44% for the 1000, 100, and 10 attomole inputs, respectively. The 10 attomole level is dominated by two outlier measurements. Removing the 6 differential pairs resulting from these two measurements reduces this difference to 24%. Given Ligo-miR's high reproducibility (5.8% intraday CV for raw fluorescence from 1500 attomoles input miRNA), measured 1.14-fold changes have a p-value of 0.069 (Independent two-sample t-test, one tailed probability). Thus, measured changes of this magnitude should be correctly resolved with 93% certainty using duplicate measurements. Larger changes of 1.5-fold should be resolved with 99% certainty. In this experiment, 40 pairs of measurements involve difficult to resolve differences of 1.5-fold or less; of these, 98% are resolved correctly. Compared to existing methods, Ligo-miR is therefore functionally capable of improving differential sensitivity when investigating subtle changes caused by either small expression changes in large numbers of cells or large expression changes in small numbers of driver cells.

### Benchmarking against RT-qPCR and microarray

To benchmark Ligo-miR EZ performance, we profiled 5 esophageal cell lines (HEEPIC, CHTRT, QHTRT, SKGT4, and OE33) and compared against Applied Biosystems TaqMan RT-qPCR and Agilent microarray. RNA was pooled for each cell line to eliminate variation from sample preparation and cell culture. Ligo-miR EZ was performed using a 26-plex probe set (Panel A), resulting in 5 total reactions. TaqMan was performed across 21 miRNA using triplicate RT and duplicate qPCR steps, resulting in 630 total qPCR reactions (S1 File, Table S5). Of these, 77 outliers were identified due to anomalous amplification curves, and 18 reactions did not result in any amplification; these 95 traces were omitted when calculating the mean C_t_s used in differential analysis (S1 File, Tables S6). The average standard deviation across all miRNA (excluding null results but including outliers) is 0.71 C_t_s, corresponding to an expression difference of 63%. We further measured miR-106b-5p for all cells on three separate days to quantify day-to-day technical variation (S1 File, Table S7). The average standard deviation of the raw C_t_s is 0.58, corresponding to a variation in miRNA expression of 48%, which is significantly higher than the equivalent measure from Ligo-miR EZ, 22%. Microarray was performed according to the manufacturer’s protocol using a single Agilent slide with 8 arrays, each containing probes for 2006 miRNA. Expression values are provided in S1 File, Table S8.

Differential expression was calculated by dividing the miRNA expression level in each cell line against OE33. The differential expression obtained using RT-qPCR is plotted against that from Ligo-miR EZ in Fig 4d and shows that the two methods agree well with a strong linear fit (gradient = 0.91 ± 0.05, *r*^2^ = 0.93). An equivalent comparison between microarray and Ligo-miR EZ is shown in Fig 4e and demonstrates good correlation (gradient = 0.84 ± 0.05, *r*^2^ = 0.92), although the lower gradient may suggest a systematic difference in expression response between the two methods. Finally, Fig 4f compares the differential expression profiles from RT-qPCR against microarray (gradient = 1.00 ± 0.04, *r*^2^ = 0.95) and also shows good agreement. Thus, all the measurement platforms obtain similar expression profiles, and Ligo-miR performs equally as well as the two established technologies. Equivalent correlation values between a variety of different sequencing and microarray platforms and RT-qPCR were previously measured to be between 0.68 and 0.92 [37]. The agreement between miRNA expression measured by Ligo-miR EZ and RT-qPCR is therefore among the highest found between the methods.

### Absolute copy number determination

**Fig 5:**
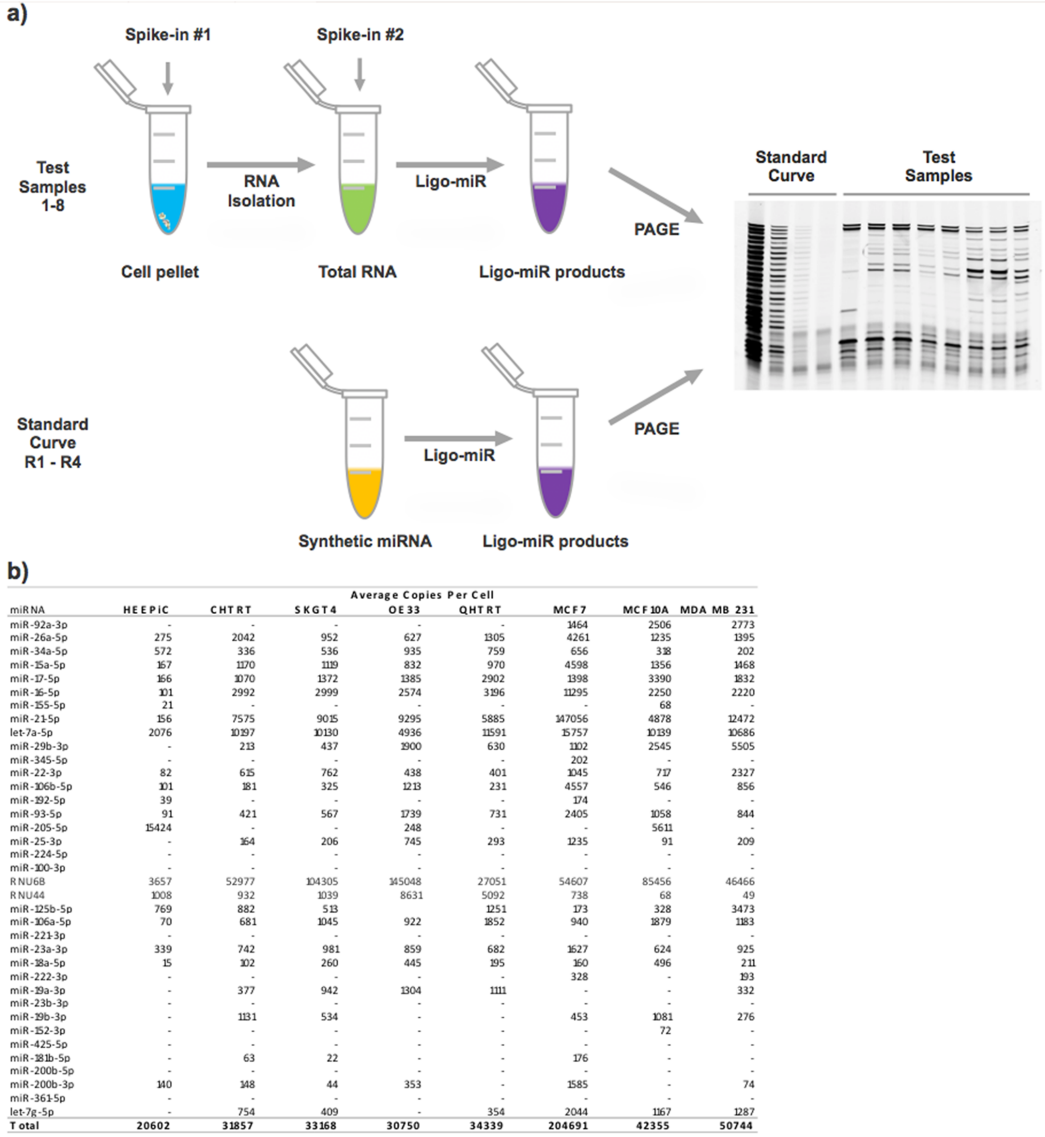
Absolute miRNA copy number using Ligo-miR EZ. **a)** Schematic illustration of Ligo-miR EZ method for determining absolute copy number by using spike-in controls and standard curve samples to normalize for reaction efficiencies and assay response. **b)** Absolute copy number profiles for 3 breast cell lines and 5 esophageal cell lines were determined using 4 overlapping Ligo-miR EZ panels.

A major strength of Ligo-miR EZ is the ease with which absolute miRNA copy numbers per cell (or copies per ng of total RNA) can be measured. Absolute copy number is determined by spiking technical controls into the cell pellet and into the various reaction master mixes to account for differences in technical efficiency (Fig 5a) and then comparing the signal against a 4 point standard curve (S1 File, Fig S9). These steps can be integrated into the analysis as a matter of routine with negligible additional effort. To demonstrate the strength and feasibility of this approach, we used Ligo-miR EZ Panels A, B, C, and D to determine miRNA copy numbers in 3 breast cell lines (MCF-10A, MDA-MB-231, and MCF-7) and 5 esophageal cell lines (HEEPIC, CHTRT, QHTRT, SKGT4, and OE33). Two separate experimental designs were employed. For the breast cell lines, a constant input of 33,333 cells was used per reaction. For the esophageal cell lines a constant input of 500 ng of total RNA was used for each reaction, akin to standard differential expression analysis, and 15 pg of total RNA per cell was assumed for all calculations.

The absolute copy numbers per cell are shown in Fig 5b. The average measurement CV (S1 File, Fig S10) across all miRNA was 30%, and, as expected, lowly expressed miRNA (<1000 copies per cell) had higher variance than highly expressed miRNA (>1000 copies). These copy number CVs are higher than the previous raw fluorescence CVs as they also include variance from the spike-in and standard curve measurements. Given a mean intra-day CV of 12% for the raw fluorescence (15–1500 attomoles), we expect 99% of absolute copy numbers to be within 50% of their true value. Lower expressed miRNAs with higher intraday CVs will have correspondingly less accurate absolute number determination. The measured copy numbers ranged from 15–147,056 copies per cell and generally fell within the expected range based on previous studies [38–40]. Oncogenic miRNA such as miR-21-5p [7,8,41–43] are highly expressed in the cancer cell lines (MCF-7, MDA-MB-231, OE33, and SKGT4) and lowly expressed in the normal cell lines (HEEPIC and MCF10A). Conversely, the tumor suppressor miR-205-5p [41–46] is significantly overexpressed in the normal cell lines compared with the cancer cell lines. However, we do not see reduced expression in the cancer cell lines for some other commonly reported tumor suppressors such as let-7a-5p [41–43,47], miR-15a-5p, and miR-16-5p. This apparent discrepancy may not be significant, however, since most of the results for miR-15a-5p and miR-16-5p come from leukemia studies [48–50] with limited work relating to solid tumors [51–56]. Total miRNA expression and the number of detected miRNA also appear to increase when comparing non-cancer and cancer cells. We note that the commonly used biological controls RNU6B and RNU44 varied widely between the samples, regardless of whether the input was constant cell number or constant total RNA, highlighting concerns from previous studies [20] over the suitability of these controls. As this miRNA expression data comes from cell lines, the trends will likely differ from that found in clinical samples.

### Differential expression analysis

Analyzing differential expression using absolute copy numbers is a powerful alternative to biological normalization that can provide layered insight into miRNA expression profiles. This method reduces bias from the assay and reduces dependency on biological controls. To explore this advantage, we determined absolute expression profiles (S1 File, Table S9) using Ligo-miR EZ Panel E on a metastatic pancreatic cancer cell line treated with 0 (G0), 1 (G1), or 10 (G10) μM of the chemotherapy drug gemcitabine. Measurements of RNA isolation yield showed that gemcitabine treatment significantly reduces the total RNA per cell and corroborates the observation that cell size shrinks with increased dosage. Ligo-miR EZ analysis was performed on each of the three samples in quadruplicate using two experimental designs, a constant total RNA input of 75 ng or a constant cell input of 10,000 cells. Overall, we can see that total miRNA and RNU44 expression levels appear to be correlated (S1 File, Fig S11) and decrease with increasing gemcitabine dosage.

**Fig 6:**
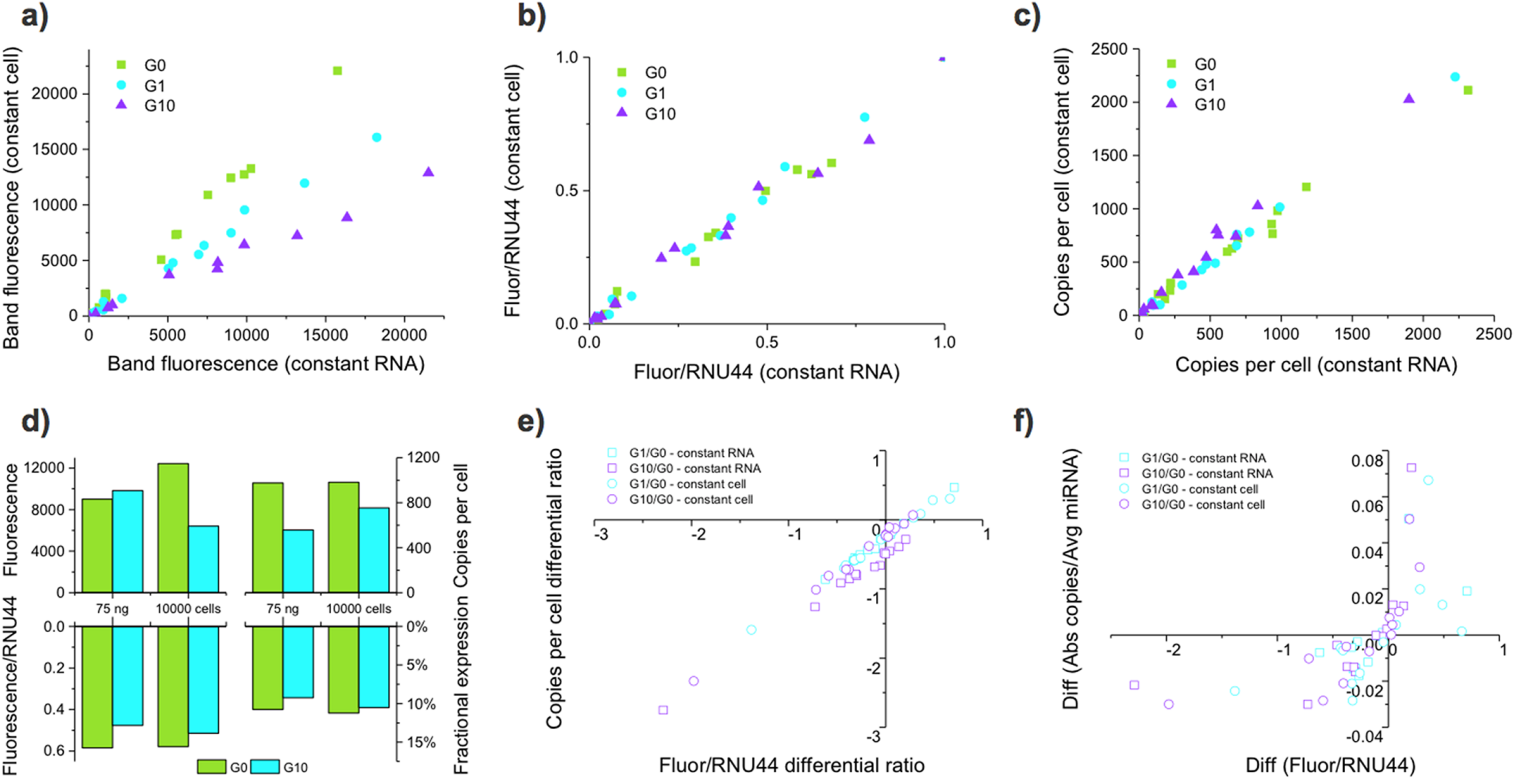
Different normalization approaches give different differential expression profiles. Ligo-miR EZ was used to profile miRNA expression of pancreatic cancer cells treated with 0, 1, or 10 μM gemcitabine. Treatment caused the cells to shrink in size and transcriptional activity. Thus, the assay was performed using either a constant total RNA input of 75 ng or a constant cell input of 10,000 cells to test the effect of experimental design on the resultant expression profiles. Each sample was measured 4 times. **a)** The fluorescent band intensities from constant RNA and constant cell inputs are correlated with a slope equal to the difference in RNA input amount. **b)** Normalization of the fluorescent intensities to RNU44 collapses the curves onto one another, demonstrating the RNU44 effectively normalizes for RNA input variation. **c)** Analysis of absolute copy number profiles also effectively normalizes the effect of RNA input variation. **d)** Analysis of miR-17 shows that the relative size of up- or down-regulation can change based on experimental design and normalization method. **e)** Differential expression ratios obtained using the RNU44 normalized fluorescence and copy number profiles are well correlated, demonstrating that both methods obtain equivalent results. **f)** The difference of each miRNA in fractional expression is plotted against the differential ratio of RNU44 normalized fluorescence to compare absolute differences in expression against ratiometric differences in expression.

Looking in greater detail, we see that the fluorescent intensities from the constant RNA input and constant cell input cases are linearly related with a slope that is equal to the difference in input amount (Fig 6a). Biological controls such as RNU44 are often used to adjust for variances in baseline transcription and input amounts [20]. Fig 6b shows that for this data set RNU44 is effective at normalizing RNA input; after RNU44 normalization, the constant cell and constant RNA input data sets collapse onto one another, eliminating differences due to experimental design. Alternatively, analysis of absolute copy number can also eliminate such differences resulting from experimental design (Fig 6c). This type of normalization is particularly important since treatments such as gemcitabine can dramatically alter the transcriptional state of the cell and generate opposing expression trends depending on experimental design.

Fig 6d shows the effect of various normalization strategies on the interpretation of miR-17-5p expression. With constant RNA input, the expression of miR-17 appears to increase with gemcitabine treatment. However, with constant cell input, miR-17 expression appears to decrease with gemcitabine treatment. Normalization of the fluorescence to RNU44 brings the constant RNA and constant cell data into alignment, with both showing a decrease in miR-17 expression with gemicitabine treatment. The RNU44 normalized data matches the copies per cell data and fractional expression data (miR-17 / total miR expression), with both showing that miR-17 expression decreases. These normalization and experimental design issues may partially explain disagreement, even in seminal work, about whether known oncogenic miRNA such as miR-17 are up- or down- regulated in tumors [7,57]. Our data show that miR-17 decreases with increasing gemcitabine dosage in terms of copies per cell, as a proportion of total miRNA expression, and with respect to RNU44. As a nucleotide analog, it is not unexpected that gemictabine would lead to reduced expression of miR-17, a known regulator of the cell cycle [58] and apoptosis [59].

Next, we calculated differential expression ratios for both the constant RNA and constant cell data sets by dividing the G_1_ and G_10_ expression levels by the G_0_ expression level. Fig 6e shows that differential ratios obtained using either the RNU44 normalized fluorescence or copies per cell data give nearly identical results. The majority of miRNA are down-regulated by gemcitabine treatment with small discrepancies seen at low differential ratios due to measurement and normalization variability. This type of differential analysis typically identifies miRNA of interest by finding those that display the highest differential ratios. In practice, the miRNA with the highest differential ratios are often those that go from undetectable to lowly expressed. As the overall expression values of these miRNA are still small, it is debatable whether these miRNA have any biological significance or effect within the cell.

Copy number profiles provide an alternative metric. Fractional expression can be determined by comparing the levels of each miRNA against the total miRNA expression within the cell. Differential comparison can be then made to examine both the ratiometric change and the absolute change. Hence, a miRNA with fractional expression that changes from 0.1% to 1% will have a smaller absolute change than a miRNA that changes from 1% to 10% despite having the same differential ratio. Fig 6f plots the differential ratio calculated using RNU44 normalized fluorescence against the absolute difference calculated from fractional expression. While the direction of change is largely preserved, the magnitude changes greatly. Some miRNA with high differential ratios have low absolute differences (e.g., miR-205-5p). Yet others with low differential ratios have high absolute differences (e.g., miR-21-5p). The greater the number of miRNA analyzed, the more accurate the fractional expression will be as the impact of individual miRNA on overall miRNA levels is reduced.

Examining miRNA in terms of absolute changes can provide layered insight into miRNA function within cells and potentially aid in identification of new biomarkers. The differences between normalizing miRNA expression to cell number, total miRNA expression, and biological controls highlight a significant challenge for miRNA measurement with biological consequences. What is more important for a miRNA's function: its expression per cell, its expression per total RNA, its expression per biological control, or its expression as a proportion of all miRNA? As we have shown, differential expressions with these normalization methods can result in misidentification of increased/decreased miRNA expression in diseased cells. Additionally, the biological relevance of ratiometric increases in expression or absolute increases in miRNA copy number per cell must be given consideration. Ligo-miR EZ provides scientists a routine method for multiplexed analysis of miRNA copy number that does not involve significant extra work, cost, or proprietary instruments. However, the convenience of fluorescent PAGE detection limits Ligo-miR EZ to 26-plex profiling and 20 copies per cell sensitivity. In future iterations, the core ligation process can be easily expanded to higher multiplexed levels, modified to further enhance sensitivity, combined with other detection systems for higher throughput, and used as a platform for broad applications in miRNA profiling.

## Supporting information

S1 File Supplementary methods. (DOCX)
