# Supplementary material for: Determination of absolute expression profiles using multiplexed miRNA analysis
Source: PLoS One. 2017 Jul 13;12(7):e0180988. doi: 10.1371/journal.pone.0180988 (PMC5509254; doi:10.1371/journal.pone.0180988)
Supplement: S1 File — (DOCX) [file pone.0180988.s001.docx]

Determination of Absolute Expression Profiles Using Multiplexed miRNA Analysis

Supplementary Methods

Yunke Song^1^, Duncan Kilburn^2^, Jee Hoon Song^3^, Yulan Cheng^3^, Christopher T Saeui^1^, Douglas G. Cheung^4^, Carlo M. Croce^4^, Kevin J Yarema^1^, Stephen J Meltzer^3^, Kelvin J Liu^2,5^, Tza-Huei Wang^1,5,6,7^

^1^ Biomedical Engineering Department, Johns Hopkins University, Baltimore, MD, USA

^2^ Circulomics Inc, Baltimore, MD, USA

^3^ Department of Medicine (GI Division) and Sidney Kimmel Comprehensive Cancer Center, The Johns Hopkins University School of Medicine, Baltimore, MD, USA

^4^ Department of Cancer Biology and Genetics, The Ohio State University, Columbus, OH, USA

^5^ Mechanical Engineering Department, Johns Hopkins University, Baltimore, MD, USA

^6^ Sidney Kimmel Comprehensive Cancer Center, Johns Hopkins University, Baltimore, MD, USA

^7^ Center of Cancer Nanotechnology Excellence, Johns Hopkins University, Baltimore, MD, USA

***Author Correspondence :** [dkilburn@circulomics.com](mailto:dkilburn@circulomics.com), thwang@jhu.edu

**Cell Line Samples**

Primary, normal, non-immortalized human esophageal epithelial cells (HEEPIC), along with esophageal cancer cell lines (SKGT4 and OE33), were purchased from ScienCell Research Laboratories (Carlsbad, California, USA) and Sigma Chemical (St Louis, Missouri, USA), respectively. The Barrett’s esophageal cell lines (CHTRT and QHTRT) were generous gifts of Dr. Peter Rabinovitch, Fred Hutchinson Cancer Center. HEEPIC and CHTRT, QHTRT cells were cultured in EpiCM-2 medium. SKGT4 and OE33 were cultured with DMEM with 10% fetal bovine serum (FBS).

Breast cell lines (MCF-7, MCF-10A, and MDA-MB-231) were purchased from ATCC (Manassas, VA). MCF-7 and MDA-MB-231 cells were grown in DMEM with 4.5 g/L glucose without L-glutamine and sodium pyruvate supplemented with 5 µL/mL penicillin/streptomycin, 1% non-essential amino-acids, and 10% FBS. MCF-10A cells were grown in DMEM/F12 supplemented with 100 µg/mL epithelial growth factor (EGF), 10 mg/mL insulin, 1 mg/mL cholera toxin, 1 mg/mL hydrocortisone, 5 µL/mL penicillin/streptomycin, and 5% horse serum. All three cell lines were grown in tissue-culture-treated T25 flasks in a 37 °C incubator with 5% CO_2_. Cells were subcultured and harvested every 5-10 days by trypsinization with 0.25%/0.02% trypsin/EDTA and immediately used for RNA extraction.

SW1990 metastatic pancreatic cancer cells (4 x 10^6^) were grown in 150 mm culture dishes with DMEM (Corning 10-013-CV) supplemented with 10% fetal bovine serum (Corning  35-011-CV) and 1% penicillin/streptomycin (Thermo Fisher Scientific  15140122).  The cells were treated with 0, 1 and 10 µM gemcitabine for 48 hours. After 2 days, the cells were harvested and pelleted (1 million cells per pellet for all samples),

**Sample Preparation**

Total RNA was isolated from the esophageal cell lines using RNeasy kits (Qiagen, Valencia, CA), combined with RNase-free DNase (Qiagen, #79254), with TRIzol reagent (Life Technologies, Carlsbad, CA) used instead of the QIAzol. The total RNA was pooled before analysis to ensure that identical samples were used for each analysis method. For RT-qPCR experiments, this total RNA was used directly. For Ligo-miR EZ analysis, the small RNA fraction was isolated from this total RNA using both Qiagen’s miRNeasy and Sigma’s mirPremier kits. The small RNA fraction was isolated directly from the breast cell lines using Qiagen’s miRNeasy and Sigma’s mirPremier kits. Pancreatic total RNA was purchased from Ambion (FirstChoice Human Pancreas RNA, catalog # AM7954).

**miRNA and Probe Synthesis**

All RNA and DNA oligonucleotides were synthesized by Integrated DNA Technologies (Coralville, IA) and re-suspended to give 100 µM stocks. All synthetic miRNA were further diluted to 1 µM concentration in TE buffer (10 mM Tris-HCl, pH = 8.0, 0.1 mM EDTA) and stored at -80 °C. These aliquots were used to prepare lower concentration stocks and discarded after single use to guard against degradation. 26-plex synthetic miRNA mixes were prepared at 20 nM from the 1µM stocks. These were also stored at -80 °C and discarded after single use. The adapters were enzymatically pre-adenylated as previously described(1) and stored at 6 µM prior to use. 3' ddC blocking prevents unwanted side reactions such as adapter concatemerization, circularization and 5' miRNA ligation. The fluorescently labeled common probes were stored at 20 µM and the discrimination probe mixes were stored at 1 µM. Unlike the miRNA stocks, we found no degradation of the DNA probes with freeze/thaw cycles and so reused the same stock multiple times.

**Ligo-miR EZ Protocol**

In the adapter ligation step, 1.5 µL sample was added to the adapter ligation master mix to create a 5 µL reaction volume with 200 nM adapter, 100 units T4 RNA ligase 2 K227Q (New England Biolabs, Ipswich, MA), 1X T T4 RNA ligase 2 K227Q buffer (New England Biolabs, Ipswich, MA), no ATP, and 25% PEG. The mixture was incubated at 25 °C for 1 hour followed by inactivation of the enzyme at 65°C for 20 minutes. Then the coding ligation master mix containing Alexa647-labeled common probe (final reaction concentration, 200 nM), up to 26x discrimination probes (final reaction concentration for each, 25 nM), and 40 units 9 °N DNA ligase (New England Biolabs, Ipswich, MA) were added to the 1st step sample to give a total volume of 13 µL. This reaction mixture was held at at 95 °C for 30 seconds to fully denature all base pairing, followed by a ligation step at 61 °C for 5 minutes. The denaturing-ligation were then cycled 50 times to amplify the fluorescently labeled, length-coded product. The DNA products were then mixed with 7 µL loading buffer and separated using 15% TBE-Urea Denaturing PAGE (Bio-Rad, Hercules, CA). The separated gels were scanned using a GE Typhoon 9410. Typically, 75 ng of total RNA was directly input into the 1st step reaction or purified to obtain small RNA which was input into the reaction. The small RNA fraction from up to 500 ng of total RNA can be used.

**Gel Image Analysis**

Handyband is a Matlab code that performs quantification of the PAGE images generated by Ligo-miR EZ. It uses a point and click interface whereby the user identifies approximate locations of Ligo-miR product bands. The program precisely locates the band midpoints, models the gel background fluorescence, and calculates the total intensity of the band profile either by numerical integration or curve-fitting. Alternatively, PAGE images can be analyzed using a combination of ImageQuant (2) (GE Healthcare, Pittsburgh, PA) and OriginPro (OriginLab, Northampton, MA). Equivalent results can be obtained with either method.

**Sample Input Effects**

High amounts of total RNA input can lead to attenuation in the coding ligation step. This is illustrated in **Fig S1** where 18-plex synthetic human miRNA were spiked into MS2 phage total RNA (Sigma-Aldrich, Cat # 10165948001). These human miRNA are not present in MS2 and enable quantification of the fractional attenuation due to total RNA inhibition. The attenuation per miRNA reflects the average of 3 separate experiments; the attenuation results are repeatable with mean CV of 7%. This data can be used to normalize the effects of background RNA attenuation.

Fig S1 Fluorescence signal attenuation of synthetic miRNA spiked into varying amounts MS2 phage total RNA. Signal attenuation increases with total RNA background. Each data series represents a specific miRNA.

**Probe Design**

All probes were designed based on miRNA sequences from miRBase 21. Thermodynamic, folding, and bioinformatic analysis were used to design length tag sequences with minimal stable secondary structure and non-specific interactions with human genome sequences. A length tag spacing algorithm was developed to evenly space Ligo-miR products with respect to the DNA sizing properties of the detection modality, leading to unique probe spacing for PAGE, CE, and single molecule analysis. This was performed by determining the sizing resolution of each detection modality as a function of DNA size and then converting uniform steps in spatial position (i.e. PAGE) or time (CE and single molecule separation) to tag length. Discrimination probe T_m_ and thermodynamic parameters were modeled using Integrated DNA Technology's OligoAnalyzer Tool. Folding was also analyzed using mfold (RNA Institute, University of Albany at State University of New York) to eliminate probe designs containing stable secondary structures such as hairpins which could inhibit hybridization or ligation. Empirical experiments were performed to determine the effects of probe T_m_, miRNA GC content, miRNA 3' base, tag length, and tag sequence on Ligo-miR EZ signal intensity (**Figs S2** **and** **S3**). A thermodynamic model was devised to account for the entropic effects of the tag length on discrimination probe T_m_. For select probes, T_m_ normalization was also performed via truncation. These parameters were combined to design 26-plex discrimination probe sets with low predicted amplification bias. During the design process, length tag sequences, recognition sequences, and the full discrimination probe sequences were screened for unwanted interactions in the human genome using tools such as Primer3 (http://bioinfo.ut.ee/primer3/) and BLAST (https://blast.ncbi.nlm.nih.gov/Blast.cgi). Five 26-plex Ligo-miR EZ panels were designed as shown in **Table S2**. Additional probe sets and miRNA were designed to test for assay specificity (**Tables S3 and S4**).

Fig S2 miRNA T_m_, GC content, and 3' base have no observable correlation to Ligo-miR signal.

Fig S3 Three sets of let-7i discrimination probes were designed. Each set had contained probes with length tags of the same sequence but varying length. Both tag sequence and length affect signal intensity. Discrimination probe length had predictable effects on Ligo-miR signal that could be thermodynamically modeled.

**Response Linearity**

Table S1 Band fluorescent intensities measured from a serial dilution of synthetic miRNAs from 10,000 attomoles to 0.5 attomoles. Data shown is plotted in Fig 2b in main manuscript. Fits demonstrate linearity of assay response.

**Reproducibility**

Fig S4 Long-term stability data showing raw band intensities from 26-plex assay on 28 separate days.

Fig S5 Intraday stability data measured via 4 x triplicate experiments, performed on 16 separate days spanning a year.

Fig S6 Raw band fluorescence from back-to-back Ligo-miR EZ experiments performed using total RNA and small RNA fractions extracted from MCF-7 cells.

Fig S7 Raw band fluorescence from back-to-back Ligo-miR EZ experiments using the small RNA fraction extracted from MCF-7 cells using either Sigma or Qiagen kits. Compared with Figure 2e, additional variation is can by the different RNA isolation chemistries employed by the two kits.. The two outliers shown in red are RNU44, which is substantially different in length than miRNA.

| **miRNA** | **A** | **B** | **C** | **D** | **E** | **miRNA sequence (5' - 3')** |
| --- | --- | --- | --- | --- | --- | --- |
| crc-4 | X | X | X | X | X | ACGAACGGCCACGAAUAGCAGA |
| crc-3 | X | X | X | X | X | GUCUACACUCCCGGCCUACUCU |
| miR-92a-3p | X | X | X | X |  | UAUUGCACUUGUCCCGGCCUGU |
| miR-26a-5p | X | X | X | X | X | UUCAAGUAAUCCAGGAUAGGCU |
| miR-34a-5p | X | X | X | X | X | UGGCAGUGUCUUAGCUGGUUGU |
| miR-15a-5p | X | X | X | X | X | UAGCAGCACAUAAUGGUUUGUG |
| miR-17-5p | X | X | X | X | X | CAAAGUGCUUACAGUGCAGGUAG |
| miR-16-5p | X | X | X | X | X | UAGCAGCACGUAAAUAUUGGCG |
| miR-155-5p | X | X | X | X |  | UUAAUGCUAAUCGUGAUAGGGGU |
| miR-21-5p | X | X | X | X | X | UAGCUUAUCAGACUGAUGUUGA |
| let-7a-5p | X | X | X | X | X | UGAGGUAGUAGGUUGUAUAGUU |
| miR-29b-3p | X | X | X | X | X | UAGCACCAUUUGAAAUCAGUGUU |
| miR-345-5p | X |  |  |  |  | GCUGACUCCUAGUCCAGGGCUC |
| miR-22-3p | X |  |  | X |  | AAGCUGCCAGUUGAAGAACUGU |
| miR-106b-5p | X |  | X | X |  | UAAAGUGCUGACAGUGCAGAU |
| miR-192-5p | X |  | X |  |  | CUGACCUAUGAAUUGACAGCC |
| miR-93-5p | X |  | X | X |  | CAAAGUGCUGUUCGUGCAGGUAG |
| miR-205-5p | X |  |  |  | X | UCCUUCAUUCCACCGGAGUCUG |
| miR-25-3p | X | X |  | X |  | CAUUGCACUUGUCUCGGUCUGA |
| miR-224-5p | X |  |  |  |  | CAAGUCACUAGUGGUUCCGUU |
| miR-100-3p | X |  |  |  |  | CAAGCUUGUAUCUAUAGGUAUG |
| RNU6B | X | X | X | X |  | GUGCUCGCUUCGGCAGCACAUAUACUAAAAUUGGAACGAUACAGAGAAGAUUAGCAUGGCCCCUGCGCAAGGAUGACACGCAAAUUCGUGAAGCGUUCCAUAUUUUU |
| cel-miR-39-3p | X | X | X | X |  | UCACCGGGUGUAAAUCAGCUUG |
| RNU44 | X | X | X | X | X | CCUGGAUGAUGAUAAGCAAAUGCUGACUGAACAUGAAGGUCUUAAUUAGCUCUAACUGACU |
| crc-2 | X | X | X | X |  | GACGCACCGCCUGGAAUGUCUA |
| crc-1 | X | X | X | X |  | GUAUAAAGAGUAGUGAUCCC |
| miR-125b-5p |  | X | X |  | X | UCCCUGAGACCCUAACUUGUGA |
| miR-106a-5p |  | X | X |  |  | AAAAGUGCUUACAGUGCAGGUAG |
| miR-221-3p |  | X | X |  | X | AGCUACAUUGUCUGCUGGGUUUC |
| miR-23a-3p |  | X |  |  |  | AUCACAUUGCCAGGGAUUUCC |
| miR-18a-5p |  | X |  |  |  | UAAGGUGCAUCUAGUGCAGAUAG |
| miR-222-3p |  | X |  |  | X | AGCUACAUCUGGCUACUGGGU |
| miR-19a-3p |  | X |  |  |  | UGUGCAAAUCUAUGCAAAACUGA |
| miR-23b-3p |  | X |  |  |  | AUCACAUUGCCAGGGAUUACC |
| miR-19b-3p |  |  | X |  |  | UGUGCAAAUCCAUGCAAAACUGA |
| miR-152-3p |  |  | X |  |  | UCAGUGCAUGACAGAACUUGG |
| miR-425-5p |  |  | X |  |  | AAUGACACGAUCACUCCCGUUGA |
| miR-181b-5p |  |  |  | X |  | AACAUUCAUUGCUGUCGGUGGGU |
| miR-200b-5p |  |  |  | X |  | CAUCUUACUGGGCAGCAUUGGA |
| miR-200b-3p |  |  |  | X |  | UAAUACUGCCUGGUAAUGAUGA |
| miR-361-5p |  |  |  | X |  | UUAUCAGAAUCUCCAGGGGUAC |
| let-7g-5p |  |  |  | X |  | UGAGGUAGUAGUUUGUACAGUU |
| miR-200c-3p |  |  |  |  | X | UAAUACUGCCGGGUAAUGAUGGA |
| miR-146a-5p |  |  |  |  | X | UGAGAACUGAAUUCCAUGGGUU |
| miR-31-5p |  |  |  |  | X | AGGCAAGAUGCUGGCAUAGCU |

Table S2 26-plex Ligo-miR Panels A, B, C, D, and E along with the miRNA sequences.

| **miRNA** | **miRNA Sequence (5' - 3')** |
| --- | --- |
| let-7a-5p | UGAGGUAGUAGGUUGUAUAGUU |
| let-7b-5p | UGAGGUAGUAGGUUGUGUGGUU |
| let-7c-5p | UGAGGUAGUAGGUUGUAUGGUU |
| let-7d-5p | AGAGGUAGUAGGUUGCAUAGUU |
| let-7e-5p | UGAGGUAGGAGGUUGUAUAGUU |
| let-7f-5p | UGAGGUAGUAGAUUGUAUAGUU |
| let-7g-5p | UGAGGUAGUAGUUUGUACAGUU |
| let-7i-5p | UGAGGUAGUAGUUUGUGCUGUU |

Table S3 let-7 family probe set miRNAs. The bases that differ among the family members are highlighted in green and red.

| **miRNA** | **miRNA Sequence (5' - 3')** |
| --- | --- |
| let-7a-1 precursor | UGGGAUGAGGUAGUAGGUUGUAUAGUUUUAGGGUCACACCCACCACUGGGAGAUAACUAUACAAUCUACUGUCUUUCCUA |

Table S4 let-7a precursor miRNA. The mature let-7a sequence is highlighted in green.

**Differential Expression Sensitivity**

Four samples (S1, S2, S3 and S4) containing 26 synthetic miRNAs at various levels were made in a manner analogous to the miRQC study(3). 4 of the 26 miRNAs were used as internal controls (Crc-1, -2, -3, -4). For each of the 22 remaining miRNAs (20 miRNA + 2 snRNA) we randomly assigned either sample 1 (S1) or sample 2 (S2) as the base sample, and for that base sample (e.g. S1) we randomly assigned the miRNA level to be 10, 100 or 1000 attomoles, with the other sample (e.g. S2) having either 30, 300 or 2000 attomoles, respectively. Thus, the differences between expression profiles in S1 and S2 are factors of 3, 3 and 2 for low, medium and high expression-level miRNAs, respectively. S1 and S2 were then titrated to generate samples S3 = 0.25*S1 + 0.75*S2 and S4 = 0.75*S1 + 0.25*S2 (**Fig S8**). The smallest ratio between two bands is the 1.14-fold difference between S3 and S2 at the 1000 attomoles level while the largest ratio is between 3-fold change between S1 and S2 at the 10 or 100 attomoles level. For unknown reasons, RNU6B, failed to be detected in all cases. The mean absolute difference between measured differential ratio and the input differential ratio was calculated and expressed as a fraction of the input differential ratio.

Fig S8 Differential sensitivity samples S1, S2, S3, and S4.

**RT-qPCR Benchmarking**

We used single tube Applied Biosystems TaqMan microRNA Assays, which include miRNA specific stem-loop reverse transcription primers, PCR primers and TaqMan probes, Applied Biosystems TaqMan MicroRNA Reverse Transcription Kit, and Bio-Rad iQ Supermix. An Applied Biosystems 7900HT Real-Time PCR System was used. 5 ng of total RNA was input into each RT reaction and performed according to the manufacturer’s protocol. The RT reactions were performed in triplicate and the cDNA from each was used for two PCR amplifications. Thus, each miRNA gives 6 Ct values, with three pairs that are independent from the RT stage. This allows us to get a good measurement of variation within a single PCR plate. Additionally, we measured one miRNA (miR-106b-5p) on three separate days to quantify day-to-day variability.

| **miRNA** | **CHTRT RT1** | **CHTRT RT1** | **CHTRT RT2** | **CHTRT RT2** | **CHTRT RT3** | **CHTRT RT3** | **HEEPIC RT1** | **HEEPIC RT1** | **HEEPIC RT2** | **HEEPIC RT2** | **HEEPIC RT3** | **HEEPIC RT3** | **OE33 RT1** | **OE33 RT1** | **OE33 RT2** | **OE33 RT2** | **OE33 RT3** | **OE33 RT3** |
| --- | --- | --- | --- | --- | --- | --- | --- | --- | --- | --- | --- | --- | --- | --- | --- | --- | --- | --- |
| RNU6B | 28.58 | 29.02 | 28.51 | 28.62 | 28.88 | 29.39 | 31.94 | 32.09 | 31.81 | 32.00 | 31.32 | 31.38 | 27.74 | 28.61 | 27.95 | 28.02 | 28.05 | 28.84 |
| miR-16 | 21.94 | 20.58 | 22.18 | 21.64 | 20.14 | 22.11 | 26.16 | 26.11 | 25.98 | 26.22 | 26.40 | 26.59 | 21.69 | 22.94 | 21.24 | 21.84 | 21.48 | 22.35 |
| mir-21 | 20.97 | 20.84 | 20.74 | 21.09 | 21.06 | 21.54 | 28.79 | 30.01 | 28.02 | 28.88 | 26.95 | 25.17 | 33.67 | 36.87 | 19.81 | 20.20 | 20.23 | 20.63 |
| miR-106b | 25.61 | 25.50 | 25.31 | 25.39 | 25.63 | 25.97 | 30.51 | 30.87 | 30.50 | 30.15 | 30.78 | 31.00 | 22.58 | 22.69 | 22.56 | 22.55 | 22.55 | 22.90 |
| miR-155 | 26.75 | 26.85 | 26.45 | 26.22 | 27.01 | 26.72 | 29.88 | 30.06 | 30.25 | 29.99 | 30.39 | 30.74 | 26.21 | 26.15 | 25.65 | 24.81 | 26.57 | 26.80 |
| miR-93 | 24.56 | 25.07 | 21.36 | 24.68 | 24.52 | 25.54 | 26.56 | 26.56 | 26.44 | 26.11 | 26.69 | 27.35 | 22.47 | 21.97 | 21.74 | 22.31 | 21.50 | 22.44 |
| RNU44 | 22.07 | 22.43 | 22.15 | 21.09 | 21.44 | 22.98 | 23.00 | 23.55 | 22.63 | 23.21 | 21.28 | 23.54 | 22.26 | 22.30 | 21.53 | 22.26 | 22.15 | 21.22 |
| miR-17 | 23.98 | 24.15 | 23.32 | 23.81 | 23.37 | 24.33 | 26.63 | 25.83 | 25.86 | 24.87 | 25.92 | 27.41 | 22.98 | 23.02 | 22.78 | 23.41 | 22.86 | 23.62 |
| miR-205 | U | U | U | 36.49 | 33.62 | U | 21.08 | 20.71 | 20.77 | 21.37 | 20.80 | 21.95 | 23.24 | 23.91 | 23.40 | 23.99 | 23.62 | 24.52 |
| let-7a | 21.21 | 21.10 | 20.55 | 20.52 | 20.55 | 21.55 | 23.84 | 23.60 | 23.77 | 23.97 | 23.87 | 24.05 | 22.16 | 22.46 | 21.39 | 21.78 | 21.69 | 22.05 |
| miR-25 | 25.58 | 25.34 | 24.90 | 24.99 | 25.22 | 25.30 | 27.88 | 28.35 | 27.62 | 25.71 | 28.19 | 28.74 | 22.92 | 23.25 | 22.65 | 22.69 | 22.93 | 23.26 |
| miR-26a | 22.65 | 23.63 | 23.10 | 23.19 | 23.41 | 24.04 | 27.10 | 27.49 | 26.68 | 26.78 | 26.94 | 27.52 | 23.98 | 24.12 | 23.72 | 23.97 | 23.26 | 24.61 |
| miR-92a | 22.93 | 22.85 | 22.20 | 22.33 | 22.81 | 23.76 | U | U | 22.80 | 21.62 | 23.16 | 24.03 | 23.70 | 23.67 | 21.35 | 23.18 | 23.01 | 23.93 |
| miR-29b | 26.62 | 26.24 | 25.95 | 26.23 | 25.69 | 26.52 | 34.93 | 35.50 | 34.82 | 35.34 | 36.05 | 35.32 | 23.27 | 23.99 | 23.67 | 23.17 | 22.81 | 23.91 |
| miR-345 | 27.27 | 27.68 | 26.74 | 27.14 | 26.20 | 27.95 | 30.29 | 30.14 | 30.45 | 30.32 | 29.63 | 30.80 | 26.56 | 25.95 | 25.98 | 25.97 | 26.30 | 27.27 |
| miR-15a | 26.22 | 26.14 | 26.00 | 26.25 | 25.64 | 26.13 | 32.67 | 28.89 | 32.42 | 30.34 | 32.62 | 33.09 | 26.38 | 26.93 | 26.51 | 26.73 | 26.42 | 26.53 |
| miR-34a | 28.00 | 28.00 | 26.84 | 27.11 | 26.66 | 27.85 | 29.19 | 29.52 | 28.92 | 28.87 | 29.39 | 29.65 | 26.98 | 26.38 | 26.20 | 26.61 | 25.98 | 26.84 |
| miR-192 | 29.73 | 29.61 | 28.48 | 29.63 | 28.04 | 29.02 | 32.99 | 34.21 | 33.34 | 34.30 | 32.60 | 33.77 | 28.67 | 29.27 | 28.05 | 28.39 | 27.31 | 28.80 |
| miR-224 | U | U | 35.48 | 35.76 | 35.18 | 33.45 | 28.82 | 28.70 | 22.91 | 27.79 | 27.06 | 27.38 | U | 35.91 | U | 36.04 | 33.60 | 36.55 |
| miR-100 | 29.44 | 29.96 | 29.10 | 29.17 | 29.14 | 29.56 | 35.38 | 33.10 | 33.96 | 31.64 | 30.14 | 32.92 | 32.97 | 32.59 | 31.87 | 31.93 | 31.83 | 29.28 |
| miR-22 | 26.72 | 27.00 | 26.59 | 26.93 | 27.28 | 28.29 | 30.58 | 29.81 | 29.79 | 30.19 | 29.85 | 30.17 | 27.20 | 27.92 | 26.93 | 27.49 | 27.40 | 27.40 |

Table S5 Ct values for individual TaqMan miRNA assays. RT reactions were run in triplicate for each miRNA and cell line (RT1, RT2, RT3). cDNA from each RT reaction was used for two qPCR reactions, making 630 qPCR reactions in total. Values highlighted in red are reactions with anomalous amplification traces. U denotes undetected.

| **miRNA** | **QHTRT RT1** | **QHTRT RT1** | **QHTRT RT2** | **QHTRT RT2** | **QHTRT RT3** | **QHTRT RT3** | **SKGT4 RT1** | **SKGT4 RT1** | **SKGT4 RT2** | **SKGT4 RT2** | **SKGT4 RT3** | **SKGT4 RT3** |
| --- | --- | --- | --- | --- | --- | --- | --- | --- | --- | --- | --- | --- |
| RNU6B | 29.30 | 29.55 | 28.78 | 28.70 | 29.30 | 29.50 | 27.53 | 28.33 | 28.02 | 28.23 | 27.83 | 28.41 |
| miR-16 | 19.40 | 21.97 | 20.19 | 21.95 | 21.58 | 21.98 | 21.56 | 21.87 | 21.70 | 21.65 | 21.35 | 22.19 |
| mir-21 | 20.95 | 21.33 | 21.32 | 20.11 | 21.15 | 21.90 | 19.44 | 19.69 | 20.44 | 20.94 | 20.47 | 21.04 |
| miR-106b | 25.97 | 25.98 | 25.36 | 25.59 | 25.40 | 25.74 | 24.78 | 24.82 | 24.38 | 24.33 | 24.58 | 25.08 |
| miR-155 | 26.34 | 26.17 | 26.16 | 26.21 | 26.78 | 27.03 | 26.57 | 26.58 | 26.24 | 24.94 | 26.70 | 27.04 |
| miR-93 | 24.58 | 24.94 | 24.87 | 24.72 | 25.02 | 25.09 | 24.02 | 24.19 | 23.54 | 23.84 | 24.22 | 24.29 |
| RNU44 | 22.59 | 23.17 | 21.98 | 23.19 | 22.70 | 21.93 | 21.73 | 22.39 | 21.88 | 22.17 | 21.82 | 22.35 |
| miR-17 | 23.06 | 23.48 | 22.93 | 22.97 | 22.63 | 23.51 | 23.19 | 22.95 | 22.64 | 23.12 | 22.30 | 23.76 |
| miR-205 | U | U | U | U | 35.24 | U | 28.99 | 27.81 | 28.86 | 29.31 | 28.73 | 30.16 |
| let-7a | 21.45 | 20.93 | 20.44 | 20.73 | 21.05 | 21.22 | 21.26 | 21.24 | 20.25 | 20.31 | 20.50 | 20.97 |
| miR-25 | 25.03 | 23.79 | 25.09 | 25.13 | 25.00 | 25.25 | 26.91 | 27.35 | 24.41 | 24.54 | 23.98 | 24.83 |
| miR-26a | 23.13 | 23.98 | 23.36 | 23.14 | 23.77 | 24.48 | 24.08 | 24.34 | 24.03 | 24.28 | 24.38 | 24.88 |
| miR-92a | 21.21 | 21.48 | 21.92 | 22.28 | 22.05 | 22.40 | 22.77 | 22.90 | 21.84 | 21.45 | 20.24 | 23.31 |
| miR-29b | 25.22 | 24.31 | 24.52 | 25.18 | 25.24 | 25.54 | 25.66 | 25.52 | 25.24 | 25.81 | 24.82 | 26.25 |
| miR-345 | 26.74 | 26.84 | 25.64 | 26.72 | 26.55 | 27.47 | 26.47 | 26.78 | 25.95 | 26.25 | 26.20 | 27.11 |
| miR-15a | 26.99 | 26.78 | 26.41 | 25.19 | 26.49 | 27.20 | 26.44 | 26.40 | 25.96 | 26.05 | 26.00 | 26.52 |
| miR-34a | 26.53 | 26.60 | 26.15 | 26.83 | 27.08 | 26.97 | 27.51 | 27.57 | 26.63 | 26.95 | 26.86 | 28.68 |
| miR-192 | 29.63 | 29.88 | 29.21 | 28.97 | 28.69 | 29.41 | 29.27 | 29.54 | 29.04 | 28.89 | 28.15 | 29.29 |
| miR-224 | U | 36.72 | U | 36.92 | 29.04 | U | 35.47 | 35.41 | 35.87 | 34.54 | 32.26 | 34.65 |
| miR-100 | 28.67 | 29.63 | 27.00 | 29.04 | 29.20 | 27.08 | 30.77 | 31.27 | 29.98 | 29.67 | 30.49 | 29.36 |
| miR-22 | 27.47 | 27.91 | 27.39 | 27.98 | 27.48 | 26.94 | 25.85 | 26.19 | 25.64 | 26.24 | 25.80 | 26.38 |

**Table S5 (continued)**

|  | **Mean Ct** | | | | |
| --- | --- | --- | --- | --- | --- |
| **miRNA** | **CHTRT** | **HEEPIC** | **OE33** | **QHTRT** | **SKGT4** |
| miR-92a-3p | 22.82 | 23.33 | 23.50 | 22.16 | 22.46 |
| miR-26a-5p | 23.34 | 27.08 | 23.94 | 23.64 | 24.33 |
| miR-34a-5p | 27.41 | 29.26 | 26.50 | 26.69 | 27.37 |
| miR-15a-5p | 26.06 | 32.23 | 26.58 | 26.77 | 26.23 |
| miR-17-5p | 23.83 | 26.45 | 23.11 | 23.10 | 23.00 |
| miR-16-5p | 21.97 | 26.24 | 21.92 | 21 .87 | 21.72 |
| miR-155-5p | 26.67 | 30.22 | 26.27 | 26.45 | 26.63 |
| miR-21-5p | 21.04 | 29.23 | 20.22 | 21.33 | 20.34 |
| let-7a-5p | 20.91 | 23.85 | 21.92 | 20.97 | 20.75 |
| miR-29b-3p | 26.21 | 35.33 | 23.47 | 25.00 | 25.55 |
| miR-345-5p | 27.16 | 30.27 | 26.34 | 26.66 | 26.46 |
| miR-22-3p | 27.13 | 30.06 | 27.39 | 27.65 | 26.02 |
| miR-106b-5p | 25.57 | - | 22.64 | 25.67 | 24.66 |
| miR-192-5p | 29.30 | 33.72 | 28.64 | 29.42 | 29.21 |
| miR-93-5p | 24.87 | 26.62 | 22.07 | 24.87 | 24.02 |
| miR-205-5p | 36.49 | 21.11 | 23.78 | - | 29.33 |
| miR-25-3p | 25.22 | 28.16 | 22.95 | 25.10 | 25.34 |
| miR-224-5p | - | 27.95 | - | - | 35.32 |
| miR-100-3p | 29.36 | - | - | 29.13 | 30.63 |
| RNU6B | 28.83 | 31.76 | 28.20 | 29.19 | 28.06 |
| RNU44 | 22.41 | 23.18 | 22.10 | 22.72 | 22.06 |

Table S6 Mean and standard deviations of qPCR expression data from Table S5. Undetected and anomalous data are excluded.

| **Sample** | **Oct 2** | **Oct 24** | **Nov 15** |
| --- | --- | --- | --- |
| CHTRT RT1 | 25.61 | 23.46 | 26.36 |
| CHTRT RT1 | 25.50 | 24.53 | 26.47 |
| CHTRT RT2 | 25.31 | 24.24 | 25.98 |
| CHTRT RT2 | 25.39 | 24.51 | 25.98 |
| CHTRT RT3 | 25.63 | 24.69 | 26.15 |
| CHTRT RT3 | 25.97 | 25.96 | 26.51 |
| HEEPIC RT1 | 30.51 | 29.87 | 31.85 |
| HEEPIC RT1 | 30.87 | 30.41 | 32.04 |
| HEEPIC RT2 | 30.50 | 30.00 | 31.19 |
| HEEPIC RT2 | 30.15 | 30.26 | 31.49 |
| HEEPIC RT3 | 30.78 | 29.87 | 31.77 |
| HEEPIC RT3 | 31.00 | 31.12 | 31.69 |
| OE33 RT1 | 22.58 | 22.53 | 23.15 |
| OE33 RT1 | 22.69 | 22.68 | 23.24 |
| OE33 RT2 | 22.56 | 22.00 | 22.96 |
| OE33 RT2 | 22.55 | 22.40 | 23.11 |
| OE33 RT3 | 22.55 | 22.66 | 23.19 |
| OE33 RT3 | 22.90 | 23.38 | 23.45 |
| QHTRT RT1 | 25.97 | 24.55 | 26.23 |
| QHTRT RT1 | 25.98 | 25.03 | 26.35 |
| QHTRT RT2 | 25.36 | 24.79 | 25.93 |
| QHTRT RT2 | 25.59 | 24.97 | 26.00 |
| QHTRT RT3 | 25.40 | 24.89 | 26.10 |
| QHTRT RT3 | 25.74 | 25.74 | 26.47 |
| SKGT4 RT1 | 24.78 | 24.17 | 24.80 |
| SKGT4 RT1 | 24.82 | 24.43 | 24.81 |
| SKGT4 RT2 | 24.38 | 23.80 | 24.49 |
| SKGT4 RT2 | 24.33 | 24.14 | 24.51 |
| SKGT4 RT3 | 24.58 | 23.99 | 24.52 |
| SKGT4 RT3 | 25.08 | 25.10 | 24.87 |

Table S7Ct values for individual TaqMan assays for miR-106b repeated on 3 different dates. On each date RT reactions were run in triplicate for each miRNA and cell line (RT1, RT2, RT3). cDNA from each RT reaction was used for two qPCR reactions.

**Microarray**

| **miRNA** | **OE33** | **HEEPIC** | **QHTRT** | **CHTRT** | **SKGT4** | **MDA-MB-231** | **MCF-7** | **MCF-10A** |
| --- | --- | --- | --- | --- | --- | --- | --- | --- |
| miR-92a-3p | 63.0 | 58.1 | 127.9 | 74.8 | 133.4 | 51.1 | 67.0 | 69.1 |
| miR-26a-5p | 113.4 | 11.5 | 104.0 | 138.7 | 130.6 | 84.1 | 225.9 | 40.4 |
| miR-34a-5p | 37.5 | 2.5 | 25.5 | 12.2 | 33.7 | 17.3 | 141.9 | 65.0 |
| miR-15a-5p | 75.2 | 0.1 | 38.4 | 51.3 | 103.2 | 54.3 | 124.6 | 25.0 |
| miR-17-5p | 201.2 | 11.5 | 195.4 | 94.1 | 293.8 | 110.6 | 118.8 | 156.8 |
| miR-16-5p | 648.3 | 11.1 | 406.5 | 360.1 | 721.4 | 341.9 | 812.4 | 179.3 |
| miR-155-5p | 48.0 | 0.1 | 32.2 | 20.8 | 46.4 | 0.1 | 0.1 | 0.1 |
| miR-21-5p | 8209.3 | 16.6 | 3064.5 | 2940.8 | 8080.6 | 3239.2 | 14020.8 | 1254.6 |
| let-7a-5p | 951.7 | 154.9 | 1174.2 | 1024.0 | 2172.3 | 1165.1 | 873.5 | 529.2 |
| miR-29b-3p | 1073.0 | 4.1 | 218.6 | 121.5 | 339.7 | 555.3 | 120.1 | 159.4 |
| miR-345-5p | 0.1 | 0.1 | 0.1 | 0.1 | 0.1 | 0.1 | 4.5 | 0.1 |
| miR-22-3p | 78.8 | 8.1 | 50.9 | 100.7 | 301.1 | 181.6 | 86.0 | 68.5 |
| miR-106b-5p | 668.2 | 0.1 | 59.6 | 52.0 | 199.8 | 120.6 | 600.1 | 55.0 |
| miR-192-5p | 0.1 | 0.1 | 0.1 | 0.1 | 0.1 | 0.1 | 12.9 | 0.1 |
| miR-93-5p | 572.1 | 11.5 | 54.9 | 44.1 | 187.2 | 101.8 | 431.2 | 61.2 |
| miR-205-5p | 399.2 | 2078.2 | 0.1 | 0.1 | 10.2 | 0.1 | 4.7 | 1786.2 |
| miR-25-3p | 457.0 | 6.0 | 60.4 | 54.6 | 160.7 | 84.6 | 315.5 | 34.8 |
| miR-224-5p | 0.1 | 0.1 | 0.1 | 0.1 | 0.1 | 23.6 | 0.1 | 8.8 |
| miR-100-3p | 0.1 | 0.1 | 0.1 | 0.1 | 0.1 | 11.6 | 0.1 | 0.1 |
| miR-125b-5p | 9.1 | 87.1 | 207.3 | 159.9 | 174.1 | 788.3 | 5.8 | 108.2 |
| miR-17-5p | 201.2 | 11.5 | 195.4 | 94.1 | 293.8 | 110.6 | 118.8 | 156.8 |
| miR-221-3p | 271.0 | 29.1 | 58.5 | 63.8 | 122.4 | 253.9 | 21.0 | 60.8 |
| miR-23a-3p | 548.9 | 154.5 | 360.4 | 296.3 | 947.6 | 373.1 | 425.9 | 162.6 |
| miR-18a-5p | 30.9 | 0.1 | 5.9 | 3.5 | 37.9 | 5.3 | 6.7 | 17.2 |
| miR-222-3p | 95.9 | 17.8 | 23.4 | 20.5 | 43.0 | 107.9 | 12.1 | 16.6 |
| miR-19a-3p | 113.8 | 0.1 | 109.7 | 72.7 | 186.5 | 49.8 | 54.7 | 67.2 |
| miR-23b-3p | 89.4 | 40.7 | 105.9 | 123.8 | 349.6 | 91.2 | 349.6 | 30.3 |
| miR-19b-3p | 258.7 | 0.1 | 424.1 | 233.1 | 467.4 | 157.0 | 153.4 | 211.1 |
| miR-152-3p | 0.1 | 0.1 | 3.2 | 3.0 | 6.8 | 0.1 | 5.2 | 0.1 |
| miR-425-5p | 33.5 | 2.5 | 13.6 | 9.9 | 22.4 | 20.1 | 52.2 | 4.2 |
| miR-181b-5p | 29.2 | 4.2 | 18.6 | 26.0 | 145.9 | 31.9 | 50.5 | 11.4 |
| miR-200b-5p | 1.7 | 0.1 | 0.1 | 0.1 | 0.1 | 0.1 | 2.0 | 0.1 |
| miR-200b-3p | 273.1 | 21.8 | 0.1 | 0.1 | 0.1 | 0.1 | 228.2 | 0.1 |
| miR-361-5p | 31.4 | 2.8 | 24.0 | 25.0 | 31.4 | 14.5 | 43.8 | 22.7 |
| let-7g-5p | 238.2 | 7.5 | 184.4 | 141.9 | 218.3 | 197.7 | 202.1 | 82.7 |
| miR-200c-3p | 531.7 | 153.5 | 0.1 | 0.1 | 85.6 | 441.8 | 112.1 | 0.1 |
| miR-146a-5p | 0.1 | 0.1 | 0.1 | 0.1 | 0.1 | 0.1 | 0.1 | 18.4 |
| miR-31-5p | 412.3 | 42.6 | 32.3 | 36.1 | 0.1 | 0.1 | 9.1 | 0.1 |

Table S8 Microarray data for all miRNA analyzed by Ligo-miR

Microarray analysis was performed by the JHMI Deep Sequencing and Microarray Core using Agilent Human miRNA Microarray Kit Release 19.0, 8x60K (G4872A, Agilent Technologies, Santa Clara, CA) following manufacturer’s protocols. This array contains 2006 human miRNAs from the miRBase Release 19.0 (URL- *http://microrna.sanger.ac.uk/sequences/*). Total RNA were isolated from ~2 x 10^5^ cells using RNeasy Mini kits (Qiagen) followed by quality checks of both total RNA and small RNA using a 2100 Bioanalyzer and software which detect 28S and 18S ribosomal RNA ratio, total RNA Integrity Number (RIN), small RNA and miRNA concentrations in the total RNA isolated. Only samples with 28S/18S>1.2, RIN>8 and detectable miRNA were used for the study.

150 ng of total RNA were first dephosphorylated with 11.2 units of calf intestine alkaline phosphatase at 37°C for 30 minutes and followed by end-labeling with pCp-Cy3 and 15 units of T4 RNA ligase using miRNA Complete Labeling and Hyb Kit (Agilent Technologies, Santa Clara, CA) at 16°C for 2 hours. Labeled samples were purified with Micro Bio-Spin 6 columns (Bio-Rad, Hercules, CA). Labeling efficiency and nucleic acid concentration were measured using Nanodrop 1000. Samples were then mixed with 10x blocking agent and 2x Hi-RPM hybridization buffer (Agilent Technologies) and hybridizations were carried out at 55°C with rotation at 20 rpm in a designated Agilent G2545A hybridization oven for 20 hours. Finally, microarrays were washed and scanned using an Agilent scanner controlled by Agilent Scan Control 7.0 software. Data were acquired with Agilent Feature Extraction 9.5.3.1 software for miRNA microarray. Two data files are generated for each array: Feature Extraction file contains signal intensities from all individual probes and GeneView file contains summarized signal intensities for each miRNA by combining intensities of replicate probes and background subtraction.

Data normalization and analysis were performed using GeneSpring GX 11 following software developer’s recommendation (Agilent). miRNA signal intensities from GeneView files were imported into software. Signal intensity from each array was quantile normalized, and all negative intensity values were surrogated to 1 before analysis.

**Absolute Copy Number Determination**

Absolute copy number was determined by spiking technical controls cel-miR-39, crc-1, crc-2, crc-3, and crc-4 into the cell pellet before extraction to account for extraction efficiency and directly before the first ligation step to account for variation in scanner and thermal cycler performance as well as ligation efficiency. A 1 - 1000 attomole synthetic miRNA serial dilution was analyzed alongside the actual samples to obtain 4-point standard curves (**Figure S9**). The absolute copy number of miRNAs per cell was calculated by normalizing the sample signal against the spike-in controls, the 4 point standard curves, and the MS2 inhibition data from **Figure S1**. 3 breast cell lines (MCF-7, MCF-10A, MDA-MB-231) and 5 esophageal cell lines (OE33, CHTRT, QHTRT, SKGT4, and HEEPIC ) were analyzed using Ligo-miR EZ Panels A, B, C, and D (**Table S2**). For the breast cell lines, a constant input of 30,000 cells was used for each reaction. For the esophageal cell lines a constant input of 500 ng of total RNA was used, akin to standard differential expression analysis, and 15 pg of total RNA per cell was assumed for calculations. Some miRNA are duplicated across the panels enabling replicate values. MCF-7 was analyzed in triplicate, meaning that for this cell line the miRNAs measured in all panels (e.g. let-7a) were measured 12 times. Similarly, MDA-MB-231 was analyzed in duplicate so the miRNAs measured in all panels were measured 8 times.

Fig S9 4 point standard curves are used to determine individual miRNA response and normalize assay bias.

Fig S10 CV of absolute copy numbers per cell plotted as a function of absolute copy number per cell. Highly expressed miRNA are determined with lower relative standard deviations. Thus, above 1000 copies per cell the mean CV is 27%. Below that number the mean CV is 34%.

**Pancreatic Cell Analysis**

Differential analysis was performed using Ligo-miR EZ Panel E (**Table S2**) on SW1990 metastatic pancreatic cancer cells treated with 0, 1 and 10 µM gemcitabine. The harvested and pelleted cells (1 million cells per pellet for all samples), were spiked with control RNA, followed by RNA isolation using the Qiagen miRNeasy kit and subsequent spiking with additional controls. We then measured the RNA concentration by UV absorbance. The cells treated with 0 µM gemcitabine (G_0_) gave a total RNA concentration of 210.8 ng/µl; the corresponding concentrations for 1 (G_1_) and 10 µM (G_10_) were 127.6 and 79 ng/µl. Given the constant cell input, we see that gemcitabine treatment significantly reduces the total RNA per cell. A repeat of this isolation from three identically prepared cell pellets gave nearly identical results of 244.3, 140 and 99 ng/µl. We diluted these samples and performed Ligo-miR EZ with either a constant total RNA input of 75 ng or a constant cell input of 10000 cells. 75 ng input for G_0_, G_1_, and G_10_ correspond to inputs of 6822, 11905, and 16835 cells, respectively; conversely, 10000 cells input corresponds to 110, 63, and 44.6 ng, respectively. These differences in experimental design are reflected in **Table S9.**

|  | **Constant RNA Input = 75 ng** | | | **Constant Cell Input = 10,000** | | |
| --- | --- | --- | --- | --- | --- | --- |
| **Treatment** | **G_0_** | **G_1_** | **G_10_** | **G_0_** | **G_1_** | **G_10_** |
| **Total RNA Input (ng)** | **75** | **75** | **75** | **109.9** | **63.0** | **44.6** |
| **Total Cells Input** | **6822** | **11905** | **16835** | **10000** | **10000** | **10000** |
| **Cell Size (pg/cell)** | **11.0** | **6.3** | **4.5** | **11.0** | **6.3** | **4.5** |
| **Fluorescence** |  |  |  |  |  |  |
| miR-34a-5p | - | - | - | - | - | - |
| miR-200c-3p | 7543 | 7303 | 8138 | 10890 | 6336 | 4224 |
| miR-17-5p | 9005 | 9013 | 9845 | 12429 | 7474 | 6412 |
| miR-16-5p | 5620 | 6954 | 8167 | 7372 | 5531 | 4824 |
| miR-125b-5p | 642 | 479 | 659 | 764 | 419 | 359 |
| miR-21-5p | 10275 | 13679 | 16359 | 13268 | 11956 | 8849 |
| let-7a-5p | 9847 | 9871 | 13194 | 12745 | 9541 | 7234 |
| miR-146a-5p | - | - | - | - | - | - |
| miR-221-3p | - | - | - | - | - | - |
| miR-31-5p | 1106 | 2097 | 1479 | 1633 | 1578 | 1022 |
| RNU44 | 15752 | 18240 | 21513 | 22073 | 16078 | 12887 |
| miR-29b-3p | 4590 | 5323 | 5088 | 5073 | 4781 | 3699 |
| miR-222-3p | 1036 | 925 | 1210 | 1975 | 1275 | 743 |
| miR-205-5p | 1092 | 922 | 348 | 1981 | 537 | 306 |
| miR-15a-5p | 5496 | 5037 | 4633 | 7313 | 4269 | 3250 |
| miR-26a-5p | 104 | 319 | 423 | 289 | 310 | 210 |
| **Copies per Cell** |  |  |  |  |  |  |
| miR-34a-5p | - | - | - | - | - | - |
| miR-200c-3p | 695 | 474 | 384 | 721 | 479 | 411 |
| miR-17-5p | 975 | 685 | 557 | 982 | 653 | 754 |
| miR-16-5p | 621 | 537 | 473 | 596 | 489 | 547 |
| miR-125b-5p | 183 | 100 | 97 | 153 | 94 | 93 |
| miR-21-5p | 2315 | 2226 | 1899 | 2112 | 2235 | 2025 |
| let-7a-5p | 933 | 689 | 681 | 856 | 759 | 742 |
| miR-146a-5p | - | - | - | - | - | - |
| miR-221-3p | - | - | - | - | - | - |
| miR-31-5p | 220 | 303 | 157 | 234 | 285 | 218 |
| RNU44 | 1177 | 990 | 835 | 1204 | 1014 | 1027 |
| miR-29b-3p | 940 | 778 | 545 | 765 | 781 | 802 |
| miR-222-3p | 135 | 92 | 86 | 198 | 125 | 98 |
| miR-205-5p | 223 | 148 | 33 | 301 | 100 | 60 |
| miR-15a-5p | 653 | 442 | 273 | 628 | 429 | 381 |
| miR-26a-5p | 37 | 27 | 23 | 26 | 32 | 22 |
| **Total – RNU44** | **7929** | **6501** | **5208** | **7573** | **6461** | **6151** |

Table S9 Fluorescence and copy number expression profiles for SW1990 cells treated with 0, 1, and 10 µM gemcitabine. Data is the average of quadruplicate measurements.

Fig S11 A linear relation is seen between RNU44 copies per cell and the total number of miRNA detected per cell when all samples are plotted together. Each data point is the average of 4 replicates.

1. Song,Y., Liu,K.J. and Wang,T. (2015) Efficient synthesis of stably adenylated DNA and RNA adapters for microRNA capture using T4 RNA ligase 1. *Nat. Publ. Gr.*, **5**, 1–8.

2. Song,Y., Liu,K.J. and Wang,T.H. (2014) Elimination of ligation dependent artifacts in T4 RNA ligase to achieve high efficiency and low bias microRNA capture. *PLoS One*, **9**.

3. Mestdagh,P., Hartmann,N., Baeriswyl,L., Andreasen,D., Bernard,N., Chen,C., Cheo,D., D’Andrade,P., DeMayo,M., Dennis,L., *et al.* (2014) Evaluation of quantitative miRNA expression platforms in the microRNA quality control (miRQC) study. *Nat. Methods*, **11**, 809–815.
